# Combinatorial functionomics identifies HDAC6-dependent molecular vulnerability of radioresistant head and neck cancer

**DOI:** 10.1186/s40164-024-00590-8

**Published:** 2025-01-12

**Authors:** Sharon Pei Yi Chan, Celestia Pei Xuan Yeo, Boon Hao Hong, Evelyn Mui Cheng Tan, Chaw Yee Beh, Eugenia Li Ling Yeo, Dennis Jun Jie Poon, Pek Lim Chu, Khee Chee Soo, Melvin Lee Kiang Chua, Edward Kai-Hua Chow

**Affiliations:** 1 Cancer Science Institute of Singapore, National University of Singapore, Singapore, Singapore; 2 Division of Medical Sciences, National Cancer Centre Singapore, Singapore, Singapore; 3 Duke-NUS Medical School, Cancer and Stem Cell Biology Programme, Singapore, Singapore; 4 Duke-NUS Medical School, Oncology Academic Programme, Singapore, Singapore; 5 Department of Head and Neck and Thoracic Cancers, Division of Radiation Oncology, National Cancer Centre Singapore, Singapore, Singapore; 6 NUS Centre for Cancer Research (N2CR), Yong Loo Lin School of Medicine, National University of Singapore, Singapore, Singapore; 7 The Institute for Digital Medicine (WisDM), Yong Loo Lin School of Medicine, National University of Singapore, Singapore, Singapore; 8 Department of Pharmacology, Yong Loo Lin School of Medicine, National University of Singapore, Singapore, Singapore; 9 Department of Biomedical Engineering, College of Design and Engineering, National University of Singapore, Singapore, Singapore

**Keywords:** Head and neck cancer, Radioresistance, Combination therapy, HDAC6 inhibition

## Abstract

**Background:**

Radiotherapy is the primary treatment modality for most head and neck cancers (HNCs). Despite the addition of chemotherapy to radiotherapy to enhance its tumoricidal effects, almost a third of HNC patients suffer from locoregional relapses. Salvage therapy options for such recurrences are limited and often suboptimal, partly owing to divergent tumor and microenvironmental factors underpinning radioresistance. In this study, we utilized a combinatorial functionomics approach, the Quadratic Phenotypic Optimization Platform (QPOP), to rationally design drug pairings that exploit the molecular fingerprint and vulnerability of established in vitro isogenic radioresistant (RR)-HNC models.

**Methods:**

A QPOP-specific protocol was applied to RR-HNC models to rank and compare all possible drug combinations from a 12-drug set comprising standard chemotherapy, small molecule inhibitors and targeted therapies specific to HNC. Drug combination efficacy was evaluated by computing combination index scores, and by measuring apoptotic response. Drug targeting was validated by western blot analyses, and the Comet assay was used to quantify DNA damage. Enhanced histone deacetylase inhibitor (HDACi) efficacy in RR models was further examined by in vivo studies, and genetic and chemical inhibition of major Class I/II HDACs. Regulatory roles of HDAC6/SP1 axis were investigated using immunoprecipitation, gel shift and ChIP-qPCR assays. Comparative transcriptomic analyses were employed to determine the prognostic significance of targeting HDAC6.

**Results:**

We report the therapeutic potential of combining panobinostat (pan-HDAC inhibitor) with AZD7762 (CHK1/2 inhibitor; AstraZeneca) or ionizing radiation (IR) to re-sensitize RR-HNC cells and showed increased DNA damage underlying enhanced synergy. We further refined this RR-specific drug combination and prioritized HDAC6 as a targetable dependency in reversing radioresistance. We provide mechanistic insights into HDAC6-mediated regulation via a crosstalk involving SP1 and oncogenic and repair genes. From two independent patient cohorts, we identified a four-gene signature that may have discriminative ability to predict for radioresistance and amenable to HDAC6 inhibition.

**Conclusion:**

We have uncovered HDAC6 as a promising molecular vulnerability that should be explored to treat RR-HNC.

**Supplementary Information:**

The online version contains supplementary material available at 10.1186/s40164-024-00590-8.

## Background

Radioresistance remains a major cause of tumor recurrence and treatment failure in a subset of head and neck cancer (HNC) patients. Approximately 30% of patients develop locoregional failure within five years after primary therapy and their prognosis is significantly attenuated [1, 2]. Resistance to radiotherapy (RT) is polymodal and associated with a diverse range of biological alterations both within the tumor and the surrounding microenvironment. Alterations in the genome and epigenome, as well as dysregulations in DNA repair and cell cycle checkpoint pathways have been shown to counteract irradiation-induced cell death and drive pro-survival signaling in tumor cells [3, 4]. The development of radiosensitizing agents or combination therapies to overcome radioresistance is therefore paramount in enhancing treatment efficacy for HNC patients. Currently, few FDA-approved radiosensitizers exist for the treatment of HNC. While incorporation of chemoradiotherapies with platinum-based compounds or taxanes have improved patient outcomes, these commonly used radiosensitizers possess inherent cytotoxic activity [5–7]. More recently, the landmark study combining cetuximab, a monoclonal antibody targeting EGFR, with radiation has demonstrated improved overall survival compared to radiation alone [8]. Since then, no new targeted agents has shown significant clinical benefit for radioresistant (RR)-HNC patients.

There is growing evidence that epigenetic-based drug combination strategies may have potential to disrupt multiple pathways simultaneously and reverse the radioresistant phenotype [9]. Epigenetic alterations, commonly observed upon DNA methylation and histone modification, are implicated in the development of radioresistance by stimulating multiple hallmarks of cancer, permitting cells to bypass the cytotoxic effects of radiation [10]. Histone deacetylases (HDACs) are transcriptional cofactors that modulate gene expression through their deacetylase activity on both histone and nonhistone proteins [11–13]. The differential expression patterns of HDACs have been reported in neoplastic tissues, such as HNC, altering gene transcription and enhancing cell proliferation [14]. Elevated HDAC1/6 expression was found to be associated with advanced clinical tumor stage, low radiosensitivity and poor prognosis in laryngeal and oral squamous cell carcinoma [15, 16]. Correspondingly, the oncogenic potential of HDACs have been detailed in the context of HNCs, and targeting HDACs in HNCs have shown to induce growth arrest, apoptosis, and autophagy, among others [17]. Preclinical and clinical studies have demonstrated antitumor efficacy of HDAC inhibitors, especially when used in combination with other therapies [18, 19]. HDAC inhibitors have also shown efficacy as radiation sensitizers across various cancer cell lines, concurrently serving as protective agents for normal cells [19, 20]. Though several early-stage clinical trials have demonstrated safety and efficacy with HDAC inhibitors and RT [21–23], no highly standardized biomarkers to predict radioresistance, or stratify patients for use of these radiosensitizing agents in combination or as monotherapy, have been established clinically. Furthermore, distinct mechanistic pathways and factors for radioresistance have been described, yet, the causal relationship between epigenetic reprogramming and recurrence post-RT is not fully understood.

Improving precision and personalized treatment modalities is critical to further enhance therapeutic ratio of RT, however, understanding how increasingly specific molecular targeted therapies are best applied in a complex signaling network is necessary to maximize drug efficacy. Rational design of drug combinations has become a promising approach to tackle the drug sensitivity and resistance problem in cancer treatment. We had previously developed a mechanism-agnostic, quantitative, phenotype-based drug screening method– Quadratic Phenotypic Optimization Platform (QPOP) that was successfully applied preclinically [24–27] and clinically [28, 29] to identify effective combinations against a range of drug resistant and relapsed/refractory cancers. Unlike conventional drug-screening approaches, platforms such as QPOP utilize small experimental datasets to interrogate the entire drug-dose search space and efficiently rank and compare all possible drug combinations for a specific disease model [30–32]. Given the highly aggressive and genetically diverse nature of HNCs, we hypothesize that application of QPOP towards an in vitro model of radioresistance can improve identification of RR-specific drug combinations. We also explored the cellular mechanisms underlying enhanced combinatorial synergy in RR models, and proposed a panel of HDAC6-related biomarkers to predict for radioresistance.

## Methods

### Cell culture

FaDu (hypopharyngeal carcinoma cell line) was obtained from American Type Culture Collection (ATCC, Manassas, VA), while HK-1 (nasopharyngeal carcinoma cell line) was donated by Professor George Tsao, University of Hong Kong [33]. FaDu cells were cultured in MEM medium (Gibco) supplemented with 10% fetal bovine serum (FBS) and penicillin/streptomycin (100U/mL). HK-1 cells were cultured in RPMI-1640 medium (Biowest) with the addition of 1% sodium pyruvate, 1% MEM Non-Essential Amino Acids solution (NEAA), 1% L-glutamate, 10% FBS, and penicillin/streptomycin (100U/mL). All cell cultures were maintained at 37 °C under a humidified atmosphere with 5% CO_2_. All cell lines used in this experiment were authenticated by ATCC cell line authentication service.

### Generation and characterization of in vitro RR-HNC models

RR FaDu and HK-1 cell lines were generated by exposing their respective parental cells to a course of fractionated 2 Gy irradiation over 45 daily treatments, five times a week, to a total dose of 90 Gy. Cells were seeded in a 175 cm^2^ flask prior to irradiation. Irradiation was delivered using a Gamma Cell^®^GC40 exactor source ^137^Cs-137 (Nordion, Canada) at a dose rate of 0.9 Gy/min. Routine media change of the irradiated cells was performed during the course of irradiation and passaged at 80% confluency. The induction of radiation resistance in the RR sublines was confirmed using clonogenic assays and characterization of its biological properties were performed using proliferation and migration assays (Fig. S1).

### Proliferation assay

FaDu and HK-1 cells were seeded at low density (500 cells/well) in 384-well plates and allowed to attach for 3 h. Cell viability was determined by CellTiter-Glo (CTG) Luminescent Cell Viability Assay (Promega) according to manufacturer’s instructions every 24 h for a total of 96 h. Values obtained in Relative Light Units (RLU) were normalized to media-only control wells and fitted to a growth curve using Prism 9 software (GraphPad).

### Migration assay

Serum-starved FaDu and HK-1 cells were plated in 6-well plates (1 × 10^6^ cells/well) and allowed to recover overnight. A sterile 200 μL pipette tip was used to make a vertical scratch in the cell monolayer, followed by two wash steps to remove debris from the edge of the scratch. Acquisition of images were performed at indicated timepoints with a 10 × phase objective using an Olympus IX71 microscope (Olympus Canada, Richmond Hill, Ontario). Wound healing capacity was determined using ImageJ wound healing tool and plotted with Prism 9 software (GraphPad).

### Determination of half-maximal inhibitory concentration (IC_50_) of drugs

Cells were seeded in 384-well plates (2500 cells/well) 24 h prior to drug treatment. Drugs were prepared in serial dilutions that ranged from 0.0001 μM to 100 μM and cells were treated in technical quadruplicates for 48 h. Cell viability was then measured using CTG assay (Promega) according to manufacturer’s instructions. IC_50_ values of individual drugs were determined using Prism 9 software (GraphPad) by fitting normalized cell viability values into sigmoidal dose–response curves.

### QPOP combinatorial drug treatment and analysis

A 12 drug-three dosages (IC_0_, IC_15_, IC_30_) QPOP was performed in this study. The drug panel included cytotoxic chemotherapy agents (docetaxel, cisplatin, carboplatin), epidermal growth factor receptor (EGFR) (cetuximab, erlotinib) and DNA damage response (DDR) targeted therapies (PARP1/2, ATM, ATR, CHK1, WEE1 inhibitors), an HDAC inhibitor (panobinostat) and a multi-tyrosine kinase inhibitor (vandetanib). Both WT and RR FaDu lines were screened against log dose concentrations of the panel of 12 drugs for 48 h to establish their respective dose–response curves and half-maximal inhibitory concentrations (IC_50_). IC_15_ and IC_30_ concentrations were intrapolated from the dose–response curves. An orthogonal array composite design (OACD) was used as previously described to determine the 155 experimental combinations necessary for sufficient factor screening and model fitting, and tested in technical duplicates [34]. Drugs were dispensed with the aid of a digital dispenser (D300e, Tecan) and cell viability was measured 48 h post drug treatment using CTG assay (Promega). The normalized cell viability scores were used as phenotypic inputs for QPOP second-order regression analysis as described by Rashid *et al.* [24]. QPOP then generates corresponding viability output values for all possible drug permutations. All two-drug combinations were subsequently ranked according to the output and parabolic response surface maps indicating interaction between any two drugs in combination were derived.

### Validation of drug combinations

To validate the top-ranked drug combinations determined by QPOP, we employed the use of mathematical models to quantify the combination effect of two drugs and provide better interpretation and assess reproducibility of QPOP-derived experimental data. In the median-effect (Chou-Talalay) method, single-drug and combination dose–response assays were performed to determine the IC_50_ values of each drug as monotherapy and in combination. The ratio of drug dosages between the two drugs was determined by the dosages used in QPOP and kept constant across the range of concentrations treated. Cell viability was then measured using CTG assay (Promega) 48 h post drug treatment and the combination index (CI) values were calculated according to the Chou-Talalay method [35]. Additionally, the Bliss independence model was used to compute the degree of drug synergy by categorizing combinations as synergistic, antagonistic or additive. Serial dilutions of either drug were prepared in deep well plates and added to cells in a 5 × 5 matrix of varying drug doses, with technical quadruplicates. Cell viability was measured after 48 h using CTG Assay (Promega). Bliss synergy scores were computed by inputting normalized cell viability values into SynergyFinder 2.0 application [36]. Based on deviation of observed and expected responses in the Bliss independence model, the drug combination interaction is classified as synergistic (> 10) or antagonistic (< − 10).

### Apoptosis assay

Following 48 h of drug treatment, cells were harvested and apoptosis levels were determined using the BD Pharmingen™ FITC Annexin V Apoptosis Detection Kit as per manufacturer’s instructions (BD Biosciences). Briefly, cells were stained with FITC Annexin V antibody and Propidium Iodide (PI) and percentage of cells undergoing apoptosis was measured by flow cytometry (BD LSRII, BD Biosciences). At least 10,000 events were analyzed for each sample. Quantification of percentage of apoptotic cells was performed using FlowJo software.

### Comet assay

Following 48 h of drug treatment, cells were harvested and mixed with 1% LMAgarose in PBS at 37 °C. Cell suspensions, adjusted to 10,000 cells/mL, were layered onto precoated microscope slides and allowed to solidify at 4 °C for 10 min. The slides were then immersed in lysis buffer (100 mM disodium EDTA, 2.5 M NaCl, 10 mM Tris–HCl, pH 10) containing 1% Triton X-100 overnight at 4 °C. The slides were subsequently placed in alkaline electrophoresis buffer (300 mM NaOH, 1 mM disodium EDTA, pH > 13) for 45 min to allow unwinding of the DNA. Single cell gel electrophoresis was conducted in the same buffer on a horizontal electrophoresis platform for 20 min at 25 V, 400 mA. Lastly, the slides were rinsed in neutralizing buffer (0.4 M Tris–HCl, pH 7.5), followed by staining with PI. Images were visualized by a confocal laser scanning microscope (LSM 880 Airy Scan, Carl Zeiss), with at least 5 images per sample and tail: head ratio was determined by the OpenComet software.

### Clonogenic assay

FaDu and HK-1 cells were seeded in a 60 mm dish and treated with 10 nM or 20 nM panobinostat for 24 h. Cells were subsequently irradiated with a series of 1, 2, and 4 Gy doses, and reseeded in 6-well plates. After 10–14 days, cells were stained with 0.05% crystal violet for 1 h. Colonies with more than 50 cells were counted with a microscope. Plating Efficiency (PE) was calculated by the ratio of the number of colonies counted and the number of cells plated. The surviving fraction (SF) was calculated by the ratio between the PE of irradiated cells and the PE of the non-irradiated cells. Prism 9 software (GraphPad) was used to generate the survival curves using the non-linear regression LQ model (Y = EXP [-(B1*X + B2*X^2)]).

### siRNA-mediated knockdown

FaDu and HK-1 cells were transfected with si-Negative Control (si-NC) or respective siRNAs (TriFECTa^®^ DsiRNA Kit, Integrated DNA Technologies, IDT, Singapore) using Lipofectamine RNAiMAX (Thermo Fisher) following the manufacturer’s instructions. For combinatorial drug dosing studies, AZD7762 (MedChemExpress, HY-10992) was added to cells 24 h post-transfection. Cell viability was measured 48 h post drug treatment using CTG assay (Promega), or cells were pelleted at the specified timepoints and used for protein extraction as described below. Knock-down efficiency was evaluated 72 h after transfection using western blot.

### Western blot

Cells were pelleted and washed with cold phosphate buffered saline (PBS) twice before being lysed in RIPA lysis buffer (ThermoFisher Scientific) containing both phosphatase (PhosSTOP; Roche) and protease inhibitors (cOmplete™ Protease Inhibitor Cocktail; Roche). The lysates were collected, centrifuged at 12,000 × g for 10 min, and respective protein concentrations were determined with a bicinchoninic acid protein (BCA) assay kit (Pierce, Iselin, NJ, USA). Equal amounts of protein lysates were resolved by SDS-PAGE, transferred onto PVDF membranes and serially stained with primary and secondary HRP-conjugated antibodies according to standard procedures. Protein bands were then detected via chemiluminescence using the ChemiDoc Imaging system (Bio-Rad). Primary antibodies used include PARP (Cell Signaling Technology, #9542), Caspase-3 (Cell Signaling Technology, #9662), cleaved Caspase-3 (Cell Signaling Technology, #9664), acetylated-lysine (Cell Signaling Technology, #9814), RAD51 (Abcam, ab133534), γ-H2A.X Ser139 (Cell Signaling Technology, #9718), c-MYC (Abcam, ab32072), p21 Waf1/Cip1 (Cell Signaling Technology, #2947), HDAC1 (Cell Signaling Technology, #5356), HDAC2 (Cell Signaling Technology, #5113), HDAC4 (Cell Signaling Technology, #7628), HDAC6 (Cell Signaling Technology, #7558), acetylated-α-tubulin Lys40 (Cell Signaling Technology, #3971), acetyl histone H3 Lys27 (Cell Signaling Technology, #8173), GAPDH (Cell Signaling Technology, #2118), and SP1 (Millipore, #07-645).

### Co-immunoprecipitation (co-IP) assay

Pelleted cells were washed with cold PBS twice, resuspended in 500μL lysis buffer (50 mM Tris, pH 7.6; 150 mM NaCl; 0.5% Triton-X100; 0.1% Nonidet P-40) containing protease inhibitors (Roche) and incubated on a rotator for 30 min at 4 °C. Cell lysates were centrifuged at 12,000 × g for 15 min, and protein concentrations were determined using BCA assay and normalized across samples. 800 µg of lysates were incubated with 5 µg anti-SP1 (Millipore, #07–645), anti-acetylated-lysine (Cell Signaling Technology, #9814) or normal rabbit IgG (Cell Signaling Technology, #2729) overnight at 4 °C. 25μL of Dynabeads™ Protein G (Invitrogen, 10003D) were then added to lysates and left for 2 h at 4 °C under rotation. After magnetic immunoprecipitation and washes, immunoprecipitates were resolved by SDS-PAGE, transferred, and blotted as described above.

### Electrophoretic mobility shift assay (EMSA)

Nuclear protein fractions were isolated using the NE-PER™ Nuclear and Cytoplasmic Extraction Kit (ThermoFisher Scientific) following manufacturer’s protocol. A non-radioactive electrophoretic-mobility shift assay (EMSA) kit (Signosis, GS-0040) was used to assess the transcription binding activity of SP1. Briefly, nuclear extract (10 μg) was incubated with 1 µl poly D (I-C), 2.0μL 5X Binding Buffer and 1.0μL of SP1 biotin-labelled probe or cold probe at 22 °C for 30 min in a thermocycler. DNA–protein complexes were electrophoresed in a 6.5% non-denaturing polyacrylamide gel and transferred to a positively charged nylon membrane. The protein-bound probe and free probe were immobilized with UV cross-linker and the biotin end-labelled probe was detected with streptavidin-HRP via chemiluminescence.

### Chromatin immunoprecipitation (ChIP) and quantitative PCR

Cells were washed with cold PBS twice, crosslinked with 1% (v/v) formaldehyde (Pierce) for 15 min and quenched with 0.125 M glycine. The fixed cells were lysed and sonicated for 15 cycles (15 s ON/30 s OFF) at 30% amplitude using EpiShear Probe Sonicator (Active Motif). 25 μg of sheared chromatin was incubated with 5 μg of antibodies overnight at 4 °C under rotation. 25μL of pre-equilibrated Dynabeads™ Protein G (Invitrogen, 10003D) was added to the ChIP reactions the next day and left to conjugate for 2 h at 4 °C. Beads were washed thoroughly, eluted in fresh elution buffer (1% SDS, 0.1 M NaHCO_3_) and de-crosslinked overnight at 65 °C. DNA samples were treated with RNase A and Proteinase K, purified with the QIAquick PCR purification kit (Qiagen), followed by qPCR analysis using the QuantiNova SYBR Green PCR Kit (Qiagen) on QuantStudio 5 Real-Time PCR Systems (Applied Biosystems) with primers described in Supplementary Information.

### In vivo drug treatment

All animal experiments were approved and performed according to SingHealth Institutional Animal Care and Use Committee (IACUC) guidelines. 5–6 weeks old male NCr nude mice were purchased from InVivos (Singapore). Mice were acclimatized for 3 days before the start of the experiment. 5 × 10^6^ WT and RR FaDu cells were resuspended in Matrigel (Corning, USA) before injecting into the flanks of NCr nude mice. Once tumors reached a minimum volume of 150mm^3^, mice were randomized into two groups (*n* = 5): Control (vehicle only), and 2.5 mg/kg panobinostat. Panobinostat was resuspended 10% DMSO + 90% corn oil, according to the manufacturer’s instructions. Treatment was administered via intraperitoneal injection (i.p.) on every alternate day for 24 or 34 days, and tumor volume and body weight were measured three times per week. Tumor volume was derived by the equation V = π/6 × L × B × H, where L is the largest superficial diameter, B is the smallest superficial diameter, and H is the height is the tumor. Tumor growth rate was calculated based on the difference between the first and the last tumor volume measured over the total number of days of treatment. All mice were sacrificed once tumors reached a maximum volume of 1500mm^3^ or experimental endpoint at 24 days or 34 days. Tumors were harvested for both RNA extraction and formalin-fixed paraffin embedded (FFPE) blocks for subsequent IHC analysis and TUNEL assay.

### Immunohistochemistry (IHC)

Tumor tissues harvested were fixed in 10% formalin for 36 h, dehydrated, and embedded in paraffin before cutting into 5 µm sections. FFPE sections were deparaffinized and rehydrated before incubating with primary antibody, anti-Ki67 (1:3000, Cell Signaling Technology, 9449) at 4 °C overnight. HRP Rabbit/Mouse (Dako, Denmark, #K5007) was used as a secondary antibody were then incubated at room temperature for 1 h. DAB chromogen (Dako, Denmark, K5007) was used to detect Ki67-positive cells. Sections were counterstained with 10X diluted haematoxylin and mounted with Organo Limonene medium (Sigma, USA, O8015). The percentage of positively stained cells was quantified using Qupath, by using optical density to select the positive cells out of the total cells in each field.

### TUNEL assay

DeadEnd™ Colorimetric TUNEL System (Promega, USA, G7130) was used to detect late apoptotic cells in the harvested tumor tissues. 5 µm FFPE sections were processed according to the manufacturer’s instructions. Sections were incubated with Terminal Deoxynucleotidyl Transferase (TdT) in a humidified chamber at 37 °C for 1 h, and then incubated with Streptavidin HRP. Apoptotic cells were stained using DAB chromogen (Dako, Denmark, K5007), counterstained with 6X diluted haematoxylin, and slides were mounted. The percentage of positively stained cells was quantified using Qupath, by using optical density to select the positive cells out of the total cells in each field.

### RNA sequencing (RNAseq)

Total RNA was extracted from cells using RNeasy Mini Kit (Qiagen, USA) according to the manufacturer’s instructions. Stranded RNAseq libraries were prepared by poly(A) mRNA isolation and NEBNext Ultra II Directional RNA Library Prep Kit for Illumina (New England BioLabs, USA). 150 bp paired-end sequencing was performed using Novaseq 6000 (Illumina, USA) with at least 50 million reads/samples. Adapter sequences and low-quality base calls were removed from the raw sequence reads using Trim Galore (v0.6.4) and Cutadapt (v2.10). Trimmed reads were then mapped to the hg38 reference genome using Spliced Transcripts Alignment to a Reference (STAR v2.6.1d) with standard settings. Gene quantification was performed with the “—quantMode GeneCounts” option in STAR.

### Differential gene expression analysis

Differential gene expression analysis was conducted using DESeq2 v1.38.3 in R, where the equation was designed to integrate the effects of cell line types, cell phenotypes, and HDAC6 status. To define genes differentially expressed in RR relative to WT cells, we applied a threshold of log_2_ fold-change (FC) greater than 0.5 and an adjusted *p*-value smaller than 0.1 to obtain significantly differentially expressed (DE) genes. The same criteria were used to select the DE genes in RR cells with or without genetic loss of HDAC6. Interaction analysis between the DE genes identified in cell phenotypes and genetic loss of HDAC6 status was used to select for RR genes that responded to siHDAC6. This comparison was based on the dysregulation direction of the FC between two set of DE genes. Only genes showing an opposite direction of FC in both sets of DE genes were chosen as potential RR genes responding to siHDAC6.

### Refinement of gene selection and geneset enrichment analysis (GSEA)

Refinement process was used to further select for genes identified from interaction analysis based on RNA abundance. This step is used to emphasize the effect of siHDAC6 in RR cells but not WT cells. Pairwise comparisons were conducted within modified and control cell types (WT: WT-control vs. WT-siHDAC6; RR: RR-control vs. RR-siHDAC6) to calculate relative changes in RNA abundance for these genes. Subsequently the ratio of relative changes between WT and RR groups were computed to quantify the magnitude of gene expression alterations. Only genes exhibiting a log_2_FC greater than 1 were retained, indicative of larger changes in the RR cells compared to the WT cells. The refinement process was conducted independently for each cell lines (FaDu and HK-1) and only the retained genes consensus in both cell lines were included for final selection. The final selection process was conducted to obtain the genes that related to radioresistant. GSEA was conducted using fgsea v1.24 in R to identify the enrichment pathways of these consensus genes in RR-siHDAC6 versus RR-control cells by using Gene Ontology (GO) library. The top 10 significantly positively enriched and negatively enriched pathways in siHDAC6-RR cells versus control-RR cells were selected.

### Generation and validation of prognostic signature

Gene expression profile and clinical information of patients in the Genomic Data Commons (GDC) The Cancer Genome Atlas (TCGA) HNC patient cohort database were retrieved from The Broad Institute’s Firehose database, selecting only patients who received RT or adjuvant RT (*n* = 261), along with their mRNA abundance data. A univariate Cox proportional hazard regression model was used to assess the association of gene expression with disease free survival (DFS) of HNC patients who received RT in GDC TCGA HNC cohort. The hazard ratio (HR) from the univariate Cox regression analysis was used to identify candidate genes associated with DFS from the dataset. Following generation of a candidate gene panel, the Kaplan–Meier method was used to assess the differences in DFS time of HNC patients from low and high-expression groups, and the log-rank test was used to determine the statistical significance of observed differences between groups.

### NCCS patient cohort

The National Cancer Centre Singapore (NCCS) cohort comprises 158 patients who were diagnosed with biopsy-proven NPC from August 2003 to November 2019 and received treatment with radiotherapy (RT) or concurrent chemotherapy with RT (CCRT), with or without induction chemotherapy (IC). RT was delivered using either intensity-modulated RT (IMRT) or image-guide RT (IGRT). In the definitive setting, IMRT/IGRT was delivered at doses of 74–78 Gy in 37–39 fractions, 57–60 Gy in 19–20 fractions, or 36.25–37.5 Gy in 5 fractions.

### NCCS tumour sampling and RNAseq

Treatment-naïve primary tumors from the NCCS patient cohort were sampled from formalin-fixed paraffin-embedded (FFPE) diagnostic biopsies following review by an expert pathologist to identify samples with at least 70% tumor cellularity. 10 sections of 10 μM thickness were obtained, and both DNA and RNA were extracted using ReliaPrep FFPE gDNA Miniprep System (Promega) and RNAprep pure FFPE Kit (Tiangen) respectively. Stranded RNAseq libraries were prepared using TruSeq RNA Exome kit (Illumina). 150 bp paired-end sequencing was performed using Novaseq 6000 (Illumina) with at least 50 million reads/sample. Both library preparation and sequencing were carried out by NovogeneAIT Genomics Singapore Pte Ltd. Adapter sequences and low quality base calls were removed from the raw sequence reads using Trim Galore (v0.6.4) and Cutadapt (v2.10). Trimmed reads were then mapped to the hg38 human reference genome using Spliced Transcripts Alignment to a Reference (STAR v2.6.1d) with standard settings. Gene quantification was performed with the “–quantMode GeneCounts” option in STAR.

### Statistical analysis

All experiments were performed in at least biological triplicates, with data presented as means ± standard deviation (SD). Student’s two-tailed *t* test was used for the comparison of two independent groups. A *P* value of < 0.05 was accepted as statistically significant. Prism 9 software (GraphPad) was used for data analysis.

## Results

### QPOP screen identifies combined HDAC and CHK1/2 inhibition to preferentially target RR FaDu cells

**Fig. 1:**
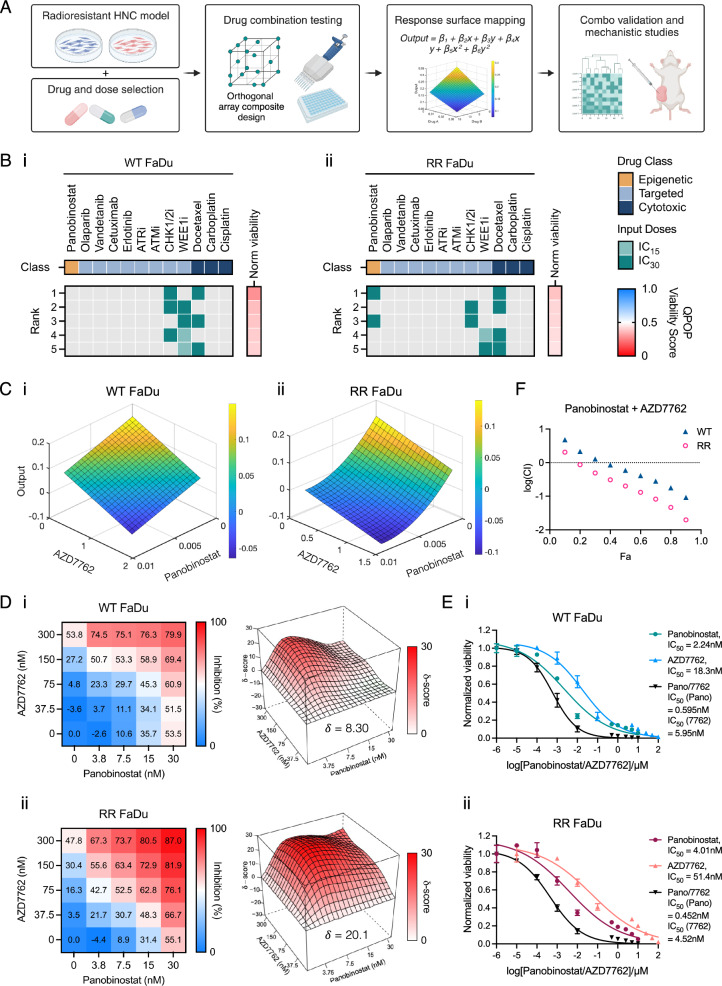
Comparative QPOP analysis identifies panobinostat and AZD7762 as top-ranking two-drug combination that preferentially targets RR FaDu cells. **a** Schematic of the study workflow from QPOP process to validation studies on top-ranking drug combinations identified by QPOP analysis. **b** Top five two-drug combinations across (i) WT and (ii) RR FaDu were ranked according to drug efficacy, defined by their corresponding QPOP viability output. Frequency of drugs were colour-mapped according to their respective concentrations (dark green: higher concentration [IC_30_]; light green: lower concentration [IC_15_]). **c** QPOP-derived parabolic response surface map depicting Pano-AZD7762 interaction in (i) WT and (ii) RR FaDu. **d** 2D dose response matrix (left) and 3D synergy distribution map (right) derived from Bliss independence model charts synergistic (red) dose regions for (i) WT and (ii) RR FaDu using inhibition (%) of cell viability as phenotypic readout. **e** Single drug and combination dose response curves of Pano and AZD7762 across (i) WT and (ii) RR FaDu, with accompanying **f** F_a_-CI (fraction affected-combination index) plot. Combination indices of log(CI) < 0 across a range of effect sizes (F_a_) is indicative of a synergistic interaction. Data presented is representative of three biological replicates

To uncover effective combination-based therapeutic sensitivities in RR-HNC, QPOP was applied to both WT and RR FaDu cells to rationally screen for drug pairings effective against RR-HNC. The overall QPOP study workflow and downstream analyses are summarized in Fig. 1a. Comparative QPOP analysis identified divergent drug sensitivities between WT and RR FaDu. All two-drug combinations were ranked based on their respective QPOP-derived normalized cell viability score and the top five drug combinations were plotted in a heatmap (Fig. 1b). A validation screen of these top-ranked combinations using the median-effect model revealed that co-treatment of panobinostat (HDAC inhibitor; Pano) and AZD7762 (CHK1/2 inhibitor) exhibited the most distinct and contrasting responses between the WT-RR pair (Fig. S2). Response surface mapping classified Pano and AZD7762 interaction to be synergistic as indicated by decreasing QPOP viability output with increasing concentrations of both drugs, though with enhanced synergy in RR FaDu (Fig. 1c). We further evaluated Pano and AZD7762 interaction using the Bliss synergy scoring model via a 5 × 5 matrix at varying drug dosages. Bliss synergy scoring revealed synergistic growth inhibition in RR (20.1, synergistic) but not in WT (8.3, additive) cells (Fig. 1d). This indicates that Pano and AZD7762 synergy is specific to the RR FaDu subline but not its parental line. From the Bliss synergy model, we prioritized the most synergistic dose ratio and further assessed combinatorial synergy at a fixed dose ratio via the median effect model. When Pano and AZD7762 were administered concurrently at a starting concentration of 100 μM in both WT and RR FaDu, there was a marked shift in the dose–response curve, and the IC_50_ of the drug combination decreased compared to when drugs were administered alone. Notably, there was a more significant shift in the combination dose–response curve in RR FaDu relative to WT (Fig. 1e). This is consistent with the F_a_-CI quantitative diagnostic plot which revealed that co-treatment led to an increasingly synergistic trend (CI < 1) with larger effect size F_a_ that is more pronounced in RR compared to WT FaDu, thereby corroborating Bliss synergy data (Fig. 1f). These results collectively demonstrate that the QPOP-optimized combination of Pano and AZD7762 preferentially targets RR FaDu cells.

### Panobinostat treatment preferentially inhibits DNA damage response in RR but not WT HNC cells

**Fig. 2:**
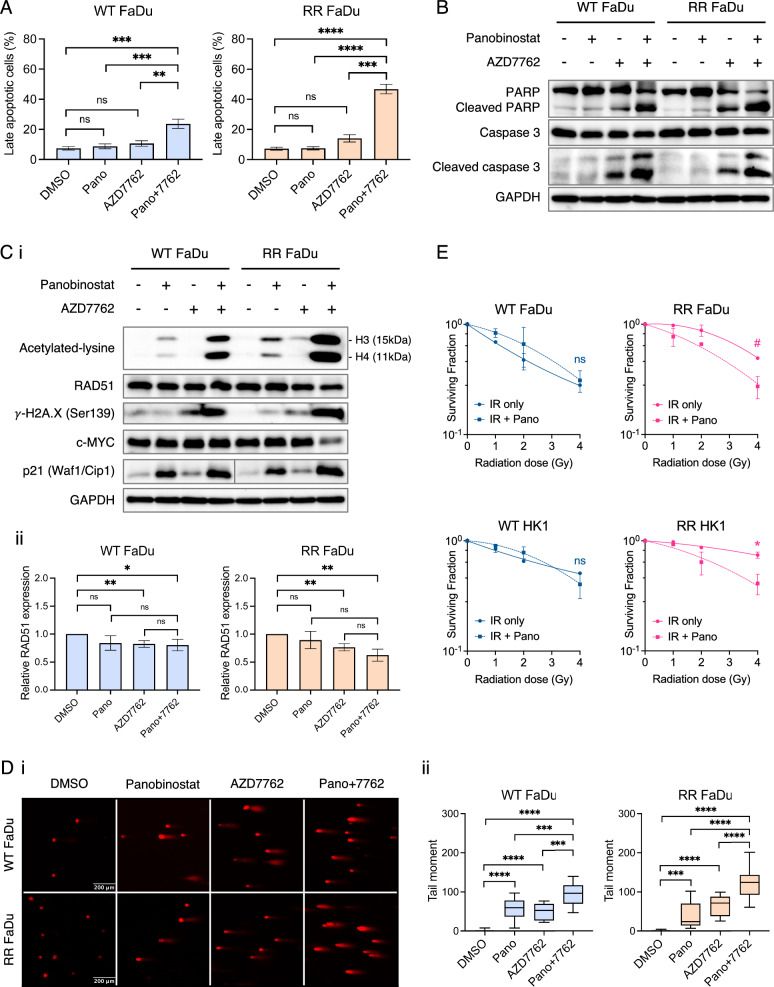
Panobinostat and AZD7762 cooperate to induce synergistic growth inhibition, up-regulation of activated DNA damage response and strand breaks, and resensitizes RR-HNC cells to RT. **a** WT and RR FaDu cells were treated for 48 h and stained with Annexin V and PI, followed by flow cytometry analysis and quantification of early versus late apoptotic population (%). **b** Representative immunoblots of cleaved caspase 3 and its downstream target cleaved PARP upon treatment with Pano and/or AZD7762, for 48 h. **c** (i) Representative immunoblots of acetylated lysine marks; RAD51 and γ-H2A.X, indicators of activated DNA damage response; tumor suppressor protein p21 (Waf1/Cip1), and transcriptional regulator c-MYC, upon treatment with Pano or AZD7762, singly or in combination, for 48 h. (ii) Quantification of RAD51 protein expression relative to GAPDH expression in (i). **d** (i) Alkaline comet assay after treatment with Pano (10 nM) and 7762 (150 nM), alone or in combination, with (ii) accompanying quantification of tail moment. Scale bars, 200 μm. *, *P* < 0.05; **, *P* < 0.01; ***, *P* < 0.001; ****, *P* < 0.0001, as compared to Pano + 7762 (2-tailed Student’s *t* test). **e** Clonogenic assay showing resensitization of RR FaDu and RR HK-1 to gamma IR with Pano (*n* = 3). #, *P* = 0.06; *, *P* < 0.05. Data presented as means ± SD of three biological replicates

We proceeded to further evaluate the efficacy of Pano and AZD7762 by characterizing their effects on apoptosis and the DNA damage response (DDR). WT and RR FaDu cells treated with a combination of 10 nM Pano and 150 nM AZD7762 rapidly underwent apoptosis, with a significantly greater fraction of cells stained positive for Annexin V/PI compared to monotherapies at 48 h (Fig. 2a). Higher synergistic cell killing was seen in RR FaDu cells treated with combination therapy compared with cells treated with Pano or AZD7762 alone (46.8 ± 3.2% vs. 7.5 ± 1.08% or 14.1 ± 2.5%, *P* < 0.0001). This effect was further confirmed by an observed increase in caspase-dependent apoptosis via cleaved caspase 3 and its downstream target cleaved PARP upon co-treatment (Fig. 2b).

Next, we analyzed treatment response by exploring the known regulatory pathways underpinning the synergistic effects of Pano and AZD7762; AZD7762 is a potent ATP-competitive checkpoint kinase inhibitor that regulates the cell cycle and DNA repair pathways [37]. Using a pan-acetylated lysine antibody, we observed that Pano treatment led to the deposition of activating acetylation marks in histones H3 and H4, which were synergistically increased when treated in combination with AZD7762 in both WT and RR FaDu. Co-treatment resulted in slight decrease in RAD51, and markedly increased histone H2A.X phosphorylation in RR FaDu, indicating increased DNA damage but decreased repair by homologous recombination (HR) specific to RR FaDu. Further analysis of cell cycle regulators revealed reactivated tumor suppressor protein p21 (Waf1/Cip1) in the WT-RR pair, but repressed oncoprotein c-MYC specific to RR FaDu with co-treatment (Fig. 2c). The Comet assay, the gold standard for DNA damage measurements, was used to further evaluate DNA damage and the DNA damage response. Significantly higher γ-H2A.X fluorescence intensity, and longer tail moment, indicative of greater extent of strand breaks, were observed in both FaDu lines with co-treatment though significantly higher in RR FaDu cells (Fig. 2d). Collectively, these results show that Pano and AZD7762 cooperate to induce synergistic growth inhibition, histone hyperacetylation, as well as up-regulation of activated DNA damage response and strand breaks most pronounced in RR FaDu.

Given that our findings strongly suggest a rational pairing of Pano with AZD7762, we further propose that similar affected pathways are shared between radiation treatment and CHK1 inhibition. Thus, we examined if Pano can resensitize RR-HNC lines to RT. Both isogenic models of FaDu and HK-1 were pre-treated with 10 nM and 20 nM Pano respectively for 24 h, before exposure to photon. Interestingly, Pano in combination with gamma IR resensitizes and reduces clonogenic survival of both RR HNC models (Fig. 2e). Exposure to Pano for 24 h before 4 Gy IR led to radiosensitization of RR HNC cells (radiosensitization ratio: FaDu = 1.77 [*P* = 0.06]; HK-1 = 1.81 [*P* = 0.041]), but not WT-HNC cells (FaDu = 0.90 [*P* = 0.60]; HK-1 = 1.26 [*P* = 0.29]). This strongly reinforces HDAC as an important pharmacological target for the resensitization of RR-HNC.

### Panobinostat demonstrates anti-tumor activity against RR HNC in vivo

**Fig. 3:**
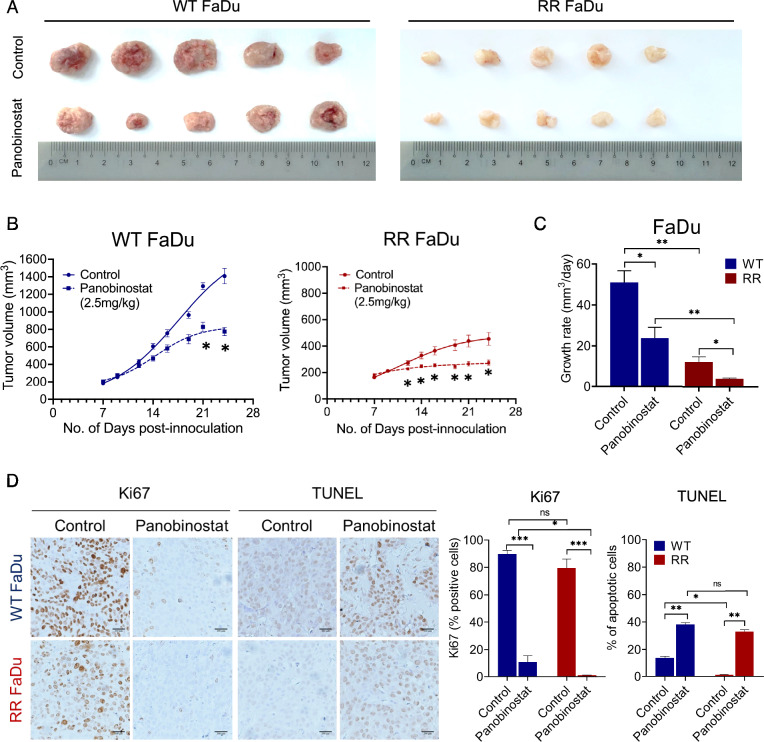
Panobinostat exhibits anti-tumor effects in in vivo models of RR-HNC. **a** Representative images and **b** tumor growth curve of WT and RR FaDu during treatment with vehicle control or 2.5 mg/kg Panobinostat. **c** Growth rate of WT and RR FaDu tumors at treatment end-point. **d** Immunohistochemistry (IHC) analysis for Ki67 proliferation marker and TUNEL assay for apoptosis detection in WT and RR FaDu tumors. Data presented as mean ± SD, *n* ≥ 5. *, *P* < 0.05; **, *P* < 0.01; ***, *P* < 0.001. All statistical analyses were performed using two-tailed Student’s *t* test

Given the preferential lethality of Pano in our RR FaDu and HK-1 cells in vitro, we proceeded to evaluate its anti-tumor efficacy in vivo. Treatment with Pano (2.5 mg/kg) impaired tumor growth in both RR and WT HNC models. We observed significant tumor growth reduction in WT FaDu tumors from Day 21 onwards, while much earlier in RR tumors at Day 12 onwards, with the significance in reduction sustained throughout the course of treatment (*P* = 0.043) (Fig. 3a-b). Moreover, while Pano treatment significantly reduced tumor growth rate in both WT and RR FaDu tumors compared to vehicle control (WT [*P* = 0.035], RR [*P* = 0.039]), we observed greater Pano/control fold-change difference in RR FaDu compared to WT (WT = 2.15; RR = 3.15) (Fig. 3c). Significant reductions in tumor volume and tumor growth rates were similarly observed in our isogenic HK-1 models (Fig. S3A-C), with greater fold-change difference observed in RR tumors. We also noted that while RR HK-1 tumors showed higher growth rate compared to WT HK-1 tumors (Fig. S3C), RR FaDu tumors showed reduced tumor growth rate compared to WT, and propose that the disparity observed in in vivo growth rates may attributed to the cell intrinsic tumorigenic properties.

In addition to tumor volume and growth rate, Pano treatment significantly suppressed proliferation in both FaDu and HK-1 isogenic models compared to vehicle treatment*.* IHC analysis of Pano-treatment tumors revealed significantly lower expression levels of Ki67 proliferative marker in both WT and RR FaDu tumors (WT [*P* = 0.0008], RR [*P* = 0.001]) as compared to tumors from respective vehicle control groups, but with a greater magnitude of change in RR tumors compared to WT (WT = 8.5-fold; RR = 73-fold) (Fig. 3d). Similarly, while a significantly lower Ki67 expression level was observed in Pano-treated HK-1 tumors compared to tumors from control groups (WT [*P* = 0.001], RR [*P* = 0.05]), we observed greater Pano/control fold-change difference in RR HK-1 compared to WT (WT = 15.5-fold; RR = 7.3-fold) (Fig. S3D). This reduction in Ki67 expression was accompanied by significantly higher levels of apoptosis in Pano-treated FaDu tumors, as shown by terminal deoxynucleotidyl transferase-mediated dUTP nick-end labeling (TUNEL) analysis. Treatment with Pano significantly induced apoptosis in both WT and RR FaDu tumors, a three-fold versus 30-fold increase in apoptotic cells compared to the vehicle-treated tumors (WT [*P* = 0.0085], RR [*P* = 0.0058]) (Fig. 3d). Moreover, a trend for increased apoptosis was observed in Pano-treated RR HK-1 tumors compared to control, though the difference was not significant (Fig. S3D). These data therefore showcased the single-agent efficacy of Pano in impairing HNC tumor growth in vivo, and its preferred lethality towards RR tumors.

### RR-specific HDACi-mediated synergy largely acts through HDAC6

**Fig. 4:**
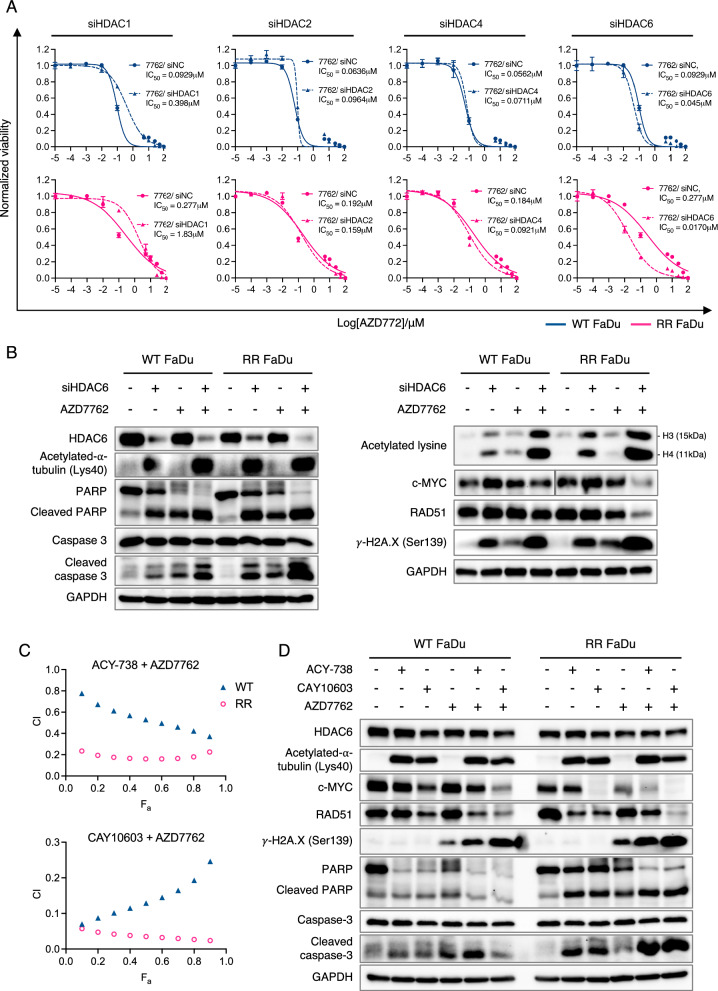
siRNA and drug inhibition assays identify HDAC6 as main contributor towards panobinostat-mediated synergy in RR models. **a** Dose–response curves of AZD7762 treated singly or in combination with siRNAs targeting HDAC1, HDAC2, HDAC4 or HDAC6 transcripts in WT (blue) or RR (pink) FaDu. **b** Representative immunoblots of HDAC6 and acetylated-α-tubulin, validating successful HDAC6 KD; cleaved caspase 3 and its downstream target cleaved PARP, apoptotic markers; RAD51 and γ -H2A.X, indicators of activated DNA damage response, acetylated histone marks, and transcriptional regulator c-MYC, in WT and RR FaDu alone or treated in combination for 48 h. **c** F_a_-CI (fraction affected-combination index) plot of AZD7762 in combination with either ACY-738 or CAY10603 in WT and RR FaDu. Combination indices of < 1 across a range of effect sizes (F_a_) is indicative of a synergistic interaction. **d** Representative immunoblots of WT and RR FaDu following treatment with either ACY-738 (10 μM) or CAY10603 (5 μM), in combination with AZD7762 (150 nM) further validating RR-specific treatment response with HDAC6 inhibition. Data presented is representative of three biological replicates

As a pan-inhibitor of HDAC enzymes, Pano induces pleiotropic mechanisms that invariably affect diverse cellular mechanisms through multiple histone and non-histone substrates [38]. Because its non-selectivity is associated with adverse events [39], the discovery of isoform-specific HDACi may offer a therapeutic advantage by minimizing toxicity profiles. We therefore proceeded to identify and evaluate the contribution of selective HDACs towards Pano-mediated synergy that is specific to our RR-HNC models. We used chemical inhibition or knockdown approach with siRNA to directly target major Class I/II HDACs and observed that siHDAC6 in combination with AZD7762 led to the greatest IC_50_ reduction in RR-HNC cells relative to WT (Figs. 4a, S4-6). Furthermore, knockdown of HDAC6 (Fig. 4b), but not other HDACs (Fig. S7), in combination with AZD7762 resulted in the strongest phenotypic change in RR cells. HDAC6 is a dual deacetylase for tubulin and histones and its functional inhibition was evidenced by upregulated levels of acetylated-⍺-tubulin and histone hyperacetylation [40]. Co-treatment with HDAC6 was also accompanied by an increase in cleaved caspase 3 and cleaved PARP apoptotic marks enhanced in RR FaDu. Notably most apparent in RR FaDu, co-treatment also led to downregulation of c-MYC, reduced HR activity and enhanced DNA damage (Fig. 4b). These findings from genetic loss-of-function studies were further supported by chemical inhibition studies using ACY-738 and CAY10603, two potent and selective inhibitors of HDAC6 demonstrating > 100 and > 200-fold selectivity, respectively, over other HDACs [41, 42]. We employed the median-effect model and demonstrated that co-treatment resulted in an increasingly synergistic trend (CI < 1) more pronounced in RR FaDu compared to WT, with larger effect size F_a_, with either ACY-738 and CAY10603 inhibitor (Fig. 4c). Combining either inhibitor with AZD7762 also led to the upregulation of apoptotic marks and the RR-specific expression profile of reduced HR efficacy and enhanced DNA damage (Fig. 4d). Therefore, deriving a unique RR-specific combinatorial treatment response via two independent mechanisms of genetic ablation or pharmacological inhibition strongly confirms that RR reversal largely acts through HDAC6.

To garner greater translational clarity on RR-specific HDAC6 dependency in vivo, we additionally performed IHC analysis of HDAC6 in WT and RR tumors. IHC analysis revealed significantly higher expression levels of HDAC6 in RR FaDu tumors compared to WT (*P* = 0.014), which was reduced by 50% upon Pano treatment *(P* = 0.006) (Fig. S3E). These findings therefore validate HDAC6 as a targetable dependency in our RR models both in vitro and in vivo.

### HDAC6 is involved in DDR control by modulating *RAD51* expression via acetylation of SP1

**Fig. 5:**
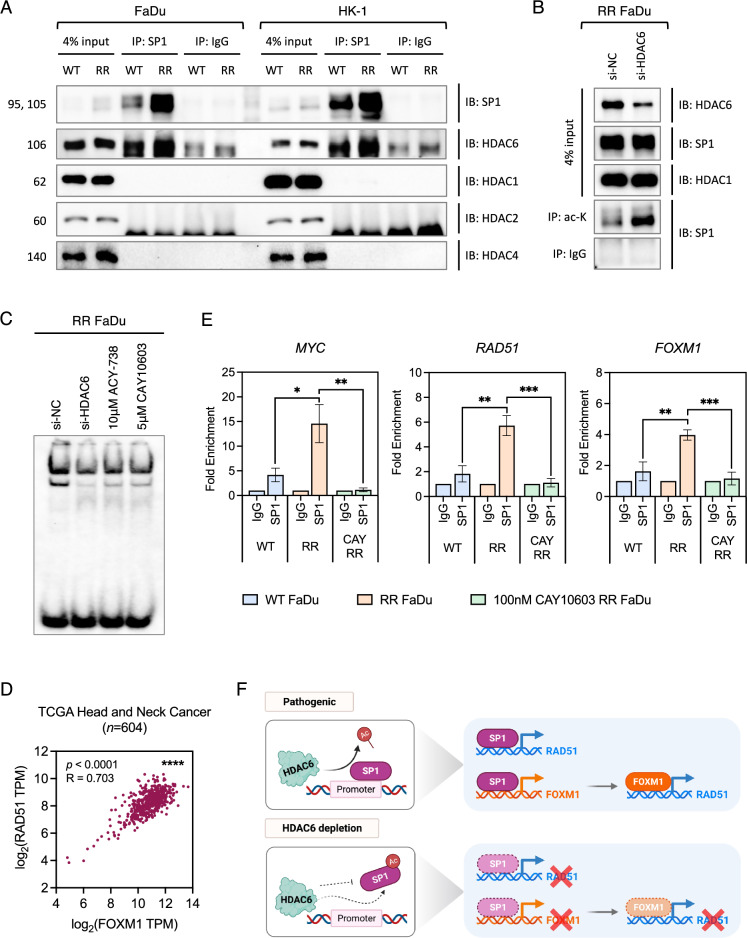
SP1 TF as an intermediate element between HDAC6 activity and RAD51 promoter inhibition. **a** Both FaDu and HK-1 cells were used for protein–protein interaction assay with anti-SP1 and normal rabbit IgG antibodies, and analyzed with immunoblotting (IB) as indicated. **b** RR FaDu cells, subjected to si-NC (negative control) or si-HDAC6, were used for protein acetylation assay with anti-acetylated-lysine and normal rabbit IgG antibodies, and analyzed with IB as indicated. **c** Analysis of SP1 DNA binding activity in RR FaDu following genetic or chemical inhibition of HDAC6. **d** Expression correlation plot of FOXM1 and RAD51 in TCGA HNC dataset (*n* = 604). The correlation coefficient (*R*) and *P* value were estimated using the Spearman correlation test. **e** ChIP and qPCR analysis of SP1 binding levels at *MYC*, *RAD51* or *FOXM1* activating promoter regions in WT and RR FaDu cells. Normal rabbit IgG antibody was used as a control for non-specific binding in ChIP assays. Data presented as mean ± SD, *n* = 3. *, *P* < 0.05; **, *P* < 0.01; ***, *P* < 0.001. All statistical analyses were performed using two-tailed Student’s *t* test. **f** Schematic illustrating how SP1 acetylation status influences the DDR signaling axis. Data presented is representative of three biological replicates

The next step was to identify an intermediate element between HDAC6 activity and the DNA damage regulatory response. A recent study in glioblastoma demonstrated that the HDAC1/2/6/SP1 pathway promoted therapeutic resistance by upregulating DNA repair pathways [43]. In Ewing sarcoma, HDAC6 modulates acetylation of SP1 and its binding to activating promoter regions [44], and a reduction in SP1 acetylation was shown to result in loss of promoter binding associated with cell cycle arrest in colon cancer [45]. HDAC6 and SP1 interaction has neither been demonstrated in head and neck cancers nor radiation resistant cancers. We hypothesized, however, that SP1 was a likely candidate to mediate HDAC6 control over DDR response. In order to test this hypothesis, we carried out co-IP studies to identify the binding partners of SP1 and demonstrated SP1 interaction with HDAC6, but not with HDAC1/2/4 (Fig. 5a). We also noted higher SP1 protein expression in both input and immunoprecipitated lanes of RR-HNC cells. In addition, knockdown of HDAC6 elevated SP1 acetylation in RR FaDu cells (Fig. 5b). These data indicate that HDAC6 is responsible for the deacetylation of SP1, which may confer radioresistance on HNC cells. Next, we performed EMSA to test the effect of HDAC6 activity on the transcriptional binding ability of SP1. Biotin-labelled hot probe incubated with WT and RR FaDu nuclear extracts resulted in the formation of two complexes, C1 and C2 (Fig. S8). Corroborating co-IP results, SP1 binding activity is elevated in RR cells relative to WT as evidenced from increased complex formation. To confirm the sequence specificity of the DNA-binding probe, competition assay was performed with the addition of unlabelled SP1 consensus probes. Mainly, C1, and to some extent, C2 disappeared after addition of the unlabelled probe, suggesting that the DNA-binding complex is specific. Genetic loss of SP1 in WT FaDu abrogated the formation of both complexes, further confirming the specificity of binding. We acknowledge that both complexes were disrupted to a lesser extent with si-SP1 RR FaDu, and theorize that this may be due to the enhanced SP1 activity in RR cells leading to oversaturation at its binding site. We additionally performed supershift assay to resolve the composition of the complexes but the supershifted complex was not clearly identified (data not shown). We proceeded to explore if the transcriptional binding ability of SP1 was influenced by HDAC6 activity in RR cells, and observed that SP1 complex formation was disrupted upon HDAC6 genetic knockdown or chemical inhibition (Fig. 5c).

To investigate if SP1 occupies and regulates the expression of DDR regulatory genes, ChIP-qPCR analysis was performed with anti-SP1 antibody in WT and RR FaDu cells. We focused on investigating expression levels of *MYC*, *RAD51* and *FOXM1* because we saw strongest phenotypic change in MYC and RAD51 levels in our preliminary data, and SP1 is also known to activate the expression of FOXM1 which in turn is a regulator for RAD51 [46]. FOXM1 is a potent oncogenic factor involved in DDR response and an enhanced FOXM1-DDR network is known to confer resistance to genotoxic agents [47]. Notably, expression correlation plot presented a strong and positive interaction between FOXM1 and RAD51 in HNC GDSC dataset (Fig. 5d), therefore signifying their relationship and functionality in similar pathways. qPCR analysis of baseline expression profile further confirmed overexpression of *MYC*, *RAD51* and *FOXM1* in RR-HNC cells relative to WT, which was lost upon treatment with CAY10603 (Fig. 5e, S9).

Our results show for the first time an HDAC6-specific epigenetic mechanism that modulates radiation resistance in HNC, with SP1 playing a central role. As illustrated in Fig. 5f, we further hypothesize that under pathogenic conditions, enhanced HDAC6 activity rapidly deacetylates SP1, allowing SP1 to autoregulate its own expression. SP1 occupy promoter regions of *RAD51*, *FOXM1* and *MYC*, which in turn further drive the expression of *RAD51* and other RR-related target genes. With HDAC6 depletion, SP1 is acetylated and its occupancy at these promoter regions is repressed, leading to downregulation of its these oncogenic and DDR targets. Overall, these results uncovered the HDAC6/SP1 axis as a putative pathway for radioresistance, and showcased the feasibility of using a selective HDAC6 inhibitor in RR-HNC.

### Characterization and validation of a four-gene prognostic signature

**Fig. 6:**
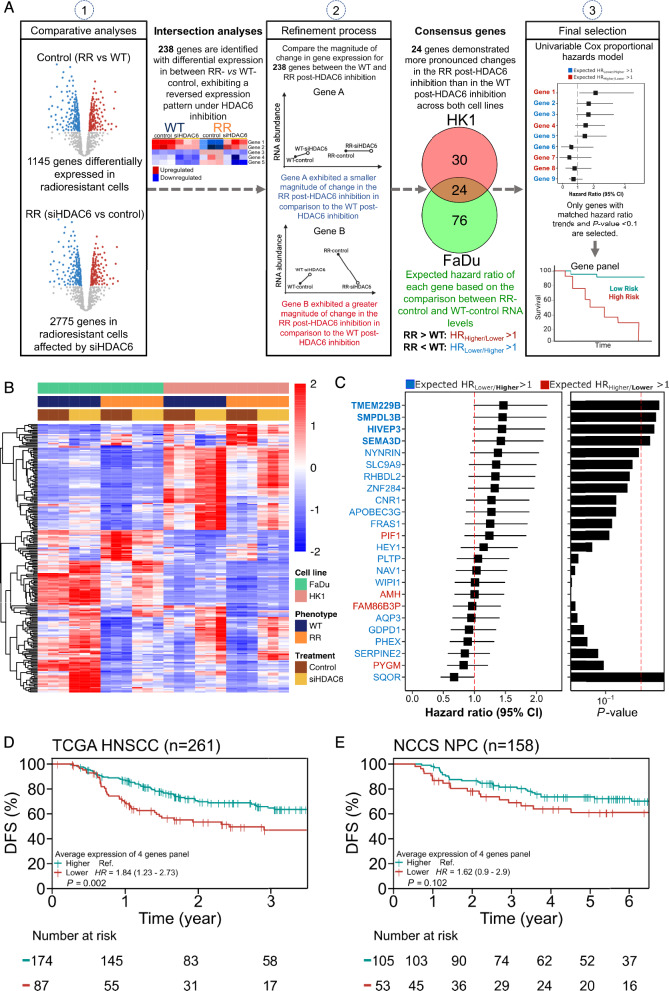
Characterization and validation of an RR-associated, HDAC6i-responsive gene panel in an independent cohort. **a** Workflow of transcriptomic analyses to identify key response genes in RR cells, correlated to RT patients in GDC TCGA head and neck cancer cohort (*n* = 261) and validated in an independent cohort. WT and RR FaDu and HK-1 cells were subjected to knockdown with siNC (negative control) or siHDAC6. **b** Heatmap depicting mRNA abundance of 238 differentially expressed genes in RR cells compared to WT and responsive to siHDAC6. **c** Forest plot established with a hazard ratio calculated through univariate Cox regression model. Blue, > 50th percentile; red, < 50th percentile as reference. **d** Kaplan–Meier curve of DFS for the four-gene signature in TCGA HNSCC dataset. **e** Kaplan–Meier curve of DFS for the four-gene signature in NCCS dataset

Apart from SP1 as a target, we also wanted to clarify the global gene changes that occur with HDAC6 inhibition in our RR models and curate a panel of candidate genes that may serve as prognostic markers. Transcriptomic profiling was performed in our isogenic RR models (FaDu and HK-1) to identify two sets of differentially expressed (DE) genes: (i) DE genes specific to RR cells compared to WT, and (ii) DE genes responsive to siHDAC6 in RR cells (Fig. 6a, S10A). A total of 1145 genes were significantly dysregulated in RR-control cells (Up: 362, Down: 783; Fig. S10B). In comparison, a greater number of genes (2775 genes) were differentially expressed with HDAC6 knockdown in RR cells (Up: 1377, Down: 1398; Fig. S10C). We derived a total of 238 intersection genes (Fig. 6b), and further refined this list to select for siHDAC6-responsive genes with greater magnitude of change in RR relative to WT cells. Among the 238 genes, 100 genes (42.0%) in FaDu, and 54 (22.7%) in HK-1, displayed more pronounced change in expression in RR compared to WT cells. A total of 24 genes (10.1%) were consensus in both cell lines (Fig. S10D), of which 4 genes (*AMH*, *FAM86B3P*, *PIF1*, and *PYGM*) were overexpressed in RR cells, while other 20 were underexpressed (Fig. S11A). Gene set enrichment analysis (GSEA) using Gene Ontology (GO) library yielded 20 significant enrichment pathways involving cell activities, cell structure, metabolic processes, chromosome structure to DNA activity and telomere activity (Fig. S11B). Notably, pathways related to chromosome, DNA activity, and telomere activity were negatively enriched in RR cells with siHDAC6, therefore lending support to our hypothesis that epigenetic reprogramming and DDR signaling axis cooperate to drive radioresistance.

To explore the potential prognostic value of this candidate panel pertinent to radioresistance, univariate Cox proportional hazard regression model was implemented to determine the association between gene expression levels and disease-free survival (DFS) outcomes in patients who received RT from the GDC TCGA Head and Neck Cancer dataset (*n* = 261). For each corresponding gene, patients in the dataset were classified into high and low-expression groups, with the median value used as the cut-off. We hypothesized that genes overexpressed in RR-control would be associated with poor prognosis in patients from high-expression group (*HR*_Higher/Lower_ > 1), while genes underexpressed in RR-control would show the opposite effect (*HR*_Lower/Higher_ > 1). A total of four genes (*TMEM229B*, *SMPDL3B*, *HIVEP3*, and *SEMA3D*) showed consistency with the hypothesis and were significantly associated with DFS (Fig. 6c). These genes may serve as candidate protective genes in RR-HNC and were used to form the prognostic signature.

We next evaluated if this four-gene signature could predict for DFS using the Kaplan–Meier method. Using GDC TCGA HNC dataset (*n* = 261), patients with low expression of this four-gene signature exhibited a poorer prognosis in DFS compared to those with higher expression (Cox proportional HR_Lower/Higher_: 1.84 [95%CI 1.23–2.73], *P* = 0.002). This indicates that patients with a lower expression score across these four genes have a higher risk of recurrence after RT. We applied the same gene panel to assess prognosis in the NCCS NPC cohort (*n* = 158). A similar trend was observed-NPC patients with low combined expression showed a poorer prognosis compared to those with a higher score (HR_Lower/Higher_: 1.62 [95% CI 0.9–2.9], *P* = 0.102). Collectively, these findings demonstrate the potential of this four-gene signature as prognostic biomarkers for risk stratification and optimization of HDAC6i-based treatment options in the context of RR-HNC.

## Discussion

RT is a DNA-targeting strategy that is the cornerstone of first-line systemic treatment of most cancers. Though its clinical applicability has positively impacted patient survival and outcomes, a major limitation of RT is that many patients develop resistance and therefore become refractory to treatment. Therefore, identifying strategies to overcome or reverse radioresistance remains crucial.

The aberrant expression and activity of HDACs has been implicated as a nexus for multiple cancer hallmarks and in mediating tumor adaptation and resistance to RT [38, 48]. Histone acetylation plays a critical role in regulating chromatin structure and gene expression– two parameters that have long been considered determinants of radioresponse. In this study, comparative QPOP screen rationally identified a HDACi-based combination that preferentially targets radioresistant sublines. Utilizing both in vitro and in vivo models, we demonstrated the single-agent efficacy of panobinostat and reinforced HDAC as an important pharmacological target for the resensitization of RR-HNC. However, we recognized the effects of panobinostat on non-specific targets and wanted to identify a clinically relevant target of translational significance. We therefore interrogated the functional significance of major Class I/II HDAC isoforms towards this RR-specific synergy. Through a series of pharmacological and molecular assays, we demonstrated HDAC6 inhibition to phenocopy the re-sensitization effects of panobinostat treatment and proposed a specific epigenetic mechanism underlying RR pathogenesis and reversal. To strengthen the clinical applicability of our findings, we uncovered a panel of common targets dysregulated in radioresistance and amenable to HDAC6 inhibition.

Dysregulation of HDAC6 deacetylase activity and HDAC6 overexpression have both been associated with therapy resistance in several solid cancers [49–52], including oral squamous cell carcinoma [53–56]. This is consistent with our current findings demonstrating HDAC6 as a promising target to overcome resistance mechanisms. The first notable observation we uncovered was that targeting HDAC6 led to strong and specific downregulation of RAD51 in RR cells. This result is in line with recent research highlighting HR pathway in enhancing radioresistance and thus its potential as a biomarker to predict response to RT [57, 58]. Our observation also builds on prior research showing that targeting RAD51 increases radiation-induced cell degradation with apoptosis [59]. We further delved into the regulatory roles of HDAC6 in RR-HNC and identified SP1 as an interactor and substrate. SP1 is a ubiquitous transcription factor crucial in cancer development [60]. It is overexpressed in most cancers and is associated with poor clinical outcomes [61, 62]. Upregulation of SP1 has been reported as a preceding event for tumor cells to gain chemotherapeutic resistance and metastatic ability in various solid tumor models [43, 63, 64]. Most notably, overexpression of SP1 was associated with tumor progression and reduced radiosensitivity in both nasopharyngeal cancer cell lines and tissue samples [65]. A few mechanisms have been proposed. In cervical cancer, SP1 has been shown to contribute to radioresistance through inhibiting G2/M phase arrest by targeting CDK1 [66]. It was also revealed that downregulation of SP1 sensitizes human glioblastoma and leukemic cells to radiation-induced DNA double-strand breaks [67, 68]. Besides its critical role in malignant progression and therapeutic resistance, the feasibility of targeting SP1 via HDAC inhibitors has also been specifically demonstrated in HNCs. Pan-HDAC inhibition was shown to decrease SP1 expression and induce cell cycle arrest and apoptosis in oral squamous cell carcinoma cell lines, consistent with reports by Shin JA *et al*. who further described reduced nuclear translocation of SP1 as a preceding event [69, 70]. Our current findings henceforth build on these studies as we identified SP1 as a downstream target of HDAC6 and described the direct effect of HDAC6i on SP1 transcriptional binding activity. We proposed that targeting HDAC6 resensitizes RR cells through the loss of SP1 binding at promoter regions of oncogenic and repair genes modulating radiation response. In this regard, pharmacological inhibition of SP1 is an attractive strategy aimed at overcoming radioresistance in HNCs or other cancer types.

Pilamycin, formerly known as Mithramycin A, is a selective SP1 inhibitor that competitively binds GC-rich DNA and globally displaces SP1 from its binding sites. The efficacy and safety of pilamycin has been assessed in Phase I/II clinical trials for advanced testicular carcinoma and refractory Ewing sarcoma, however its utility has been hampered by dose-limiting toxicity [71, 72]. Nonetheless, several novel mithramycin analogues, such as EC-8042, have been designed with enhanced anti-tumor activity and improved safety profile [73]. On the other hand, HDAC6 is recognized as a privileged target because of its unique structural and physiological functions [74]. The anti-tumor effects of two selective HDAC6 inhibitors, ricolinostat and citarinostat, have been investigated clinically as a combination-based regimen and demonstrated good response in relapsed multiple myeloma [75, 76]. Interestingly, an increasing number of studies, including preclinical models, have reported the immunoregulatory effect of HDAC6 inhibitors on cancer suppression and prolonged survival with no significant toxicity [77, 78]. Nevertheless, there is limited investigation of the effects of HDAC6 inhibitors on radioresponse, especially in treating HNC. In the present study, we described a resensitization effect on RR models that is centred around inhibition of HDAC6 activity. For greater translatability, we verified our findings with two potent and selective HDAC6 inhibitors, ACY-738 and CAY10603. It would also be interesting to explore if the radiosensitizing effects of HDAC6 inhibitors are universal in other types of cancers; its mechanism in cancers beyond HNC warrants extensive investigation.

SP1 has been shown to be involved in multiple cellular functions by regulating its downstream targets, among which are key oncogenes and tumor suppressors [79]. In this study, we proposed that HNC cells can hijack the transcriptional activity of SP1 via protein deacetylation by HDAC6. We demonstrated that SP1 occupies and regulates the expression of *RAD51*, *MYC* and *FOXM1* to trigger radiation resistance. These findings are in line with prior research which uncovered SP1 as a candidate transcription factor involved in the regulation of RAD51 in primary and recurrent glioblastoma [80]. In that study, only HDAC6 inhibition could downregulate RAD51 expression through SP1-mediated transcriptional regulation. This therefore corroborates our present findings from ChIP assays showcasing RAD51 as the most influenced DDR gene following CAY10603 treatment. Whereas, SP1 is also known to activate the expression of *FOXM1* which in turn is a regulator for *RAD51* [46]. FOXM1 is a potent oncogenic factor that has been shown to be involved in DDR response and DNA replication, and an enhanced FOXM1-DDR network can confer resistance to genotoxic agents [47]. Previously, SP1 was also identified to have the ability to bind the NHE III_1_ of the *c-MYC* promoter which controls ~ 90% of *c-MYC* transcription [81, 82]. Collectively, we have identified three key players which are targeted by the HDAC6/SP1 axis. We acknowledge a larger interplay of molecular players in this signaling axis and future work will focus on delineating other potential interactors by ChIP-seq or global proteomic analysis of acetylated proteins. We can also further verify our HDAC6-DDR signaling hypothesis by performing SP1 ChIP-seq on HDAC6 overexpressed WT-HNC cells and assess if SP1 occupancy at DDR gene promoters is increased.

The functionality of SP1, such as transcriptional activity, DNA binding, and cofactor recruitment, are tightly controlled by the only known acetylated residue, K703, which resides in its DNA-binding domain. Waby *et al.* further demonstrated that SP1 acetylation at lysine-703 releases this transcription factor from specific promoter targets [45]. Indeed, we demonstrated that HDAC6 modulates the acetylation status of SP1 and observed increased SP1 lysine acetylation after knockdown of HDAC6. Inhibition of HDAC6 acetylates and releases SP1, resulting in delayed DSB repair and the sensitization of HNC cells to RT. It will be worthy to interrogate if loss of SP1 affinity to its promoter regions by HDAC6 inhibition might be due to the acetylation of this only known residue. To garner greater mechanistic clarity, we suggest to carry out SP1 ChIP assay of WT FaDu transfected with or without acetylation-dead K703A. This will be followed by qPCR analysis of our DDR genes of interest such as *RAD51* and *FOXM1* to verify the functional significance of this lysine residue on the DDR signaling network.

Nevertheless, our study contained several limitations. We recognize that our research was based upon two established RR cell line models and therefore excludes the phenotypic and functional heterogeneity that clinical HNC samples usually present. Unfortunately, it is not feasible and impractical to perform parallel QPOP drug screens in ex vivo matched patient samples, nor perform serial QPOP analyses and follow tumor evolution from naïve to resistant state. Nonetheless, we have consistently shown HDAC-dependent molecular vulnerability in several aspects of our work, from our in vitro models to in vivo studies and independent validation of HDAC6-dependent four-gene signature in two patient cohorts. To further verify HDAC6 as a targetable dependency, we will follow up with HDAC6 and/ or ac-SP1 immunohistochemistry staining in archival FFPE tissues from patients who are RT-responders versus those who are RT-nonresponders. Further evaluations can be made as to whether HDAC6-dependency is subtype or stages-specific, or generalizable to other radioresistant cancers.

## Conclusions

We applied QPOP towards established in vitro models of radioresistance and identified an epigenetic-based therapeutic strategy that resensitized RR-HNC cell lines to DNA damage inducers, including RT. We showed that panobinostat, a pan-HDAC inhibitor, exploits a HDAC-dependent molecular vulnerability that was unique in our RR-HNC models, both in vitro and in vivo. Mechanistically, we demonstrated a critical role of HDAC6 in radioresistance that can be therapeutically exploited and further elucidated the pathological role of the HDAC6-SP1 axis in the context of HNC radioresistance. Extending our findings to a translational angle, we identified a four-gene signature that may predict HDAC6-related radioresistance in HNC and serve as a potential biomarker for HDAC-based therapy. Given the urgency to derive more actionable drug targets to restore RT treatment sensitivity, work here showcased the utility of a combinatorial functional precision medicine approach in identifying therapeutic vulnerabilities and their potential companion biomarkers in RR-HNC patients.

## Supplementary Information

Additional file 1 (Fig. S1 Characterization of in vitro isogenic models of radioresistance generated from serial irradiation method. **a** Survival curve of WT and RR FaDu and HK-1 cells following increasing doses of 0 to 4 Gy X-irradiation. **b** Growth curve of WT and RR FaDu and HK-1 cells derived from CTG luminescence values taken at the indicated timepoints. **c** Wound scratch assay performed on WT and RR FaDu cells with (i) representative images and accompanying quantification of (ii) area of wound closure (%), rate of migration (μM/h) and scratch width (μM). **d** Wound scratch assay performed on WT and RR HK-1 cells with (i) representative images and accompanying quantification of (ii) area of wound closure (%), rate of migration (μM/h) and scratch width (μM). All data presented as mean ± SD of three biological replicates. *, *P* < 0.05; **, *P* < 0.01; ***, *P* < 0.001; ****, *P* < 0.0001. All statistical analyses were performed using two-tailed Student’s *t* test.)

Additional file 2 (Fig. S2 Validation screen of top five QPOP-ranked drug combinations. **a** Single drug and combination dose response curves of QPOP-derived top-ranking drug pairs across (i) WT and (ii) RR FaDu, with accompanying **b** F_a_-CI (fraction affected-combination index) plot. Combination indices of log(CI) <0 across a range of effect sizes (F_a_) is indicative of a synergistic interaction. Data presented as means ± SD of two technical replicates.)

Additional file 3 (Fig. S3 Panobinostat exhibits anti-tumors effects in in vivo models of RR-HNC. **a** Representative tumor images, and **b** tumor growth curve of WT and RR HK-1 during treatment for 34 days with vehicle control or 2.5 mg/kg Pano. Data presented as mean ± SD, *n* ≥ 5. *, *P* < 0.05. **c **Growth rate of WT and RR HK-1 tumors vehicle control and treated tumors at treatment end-point. Data presented as mean ± SD, *n* ≥ 5. *, *P* < 0.05; **, *P* < 0.01. **d** Immunohistochemistry (IHC) analysis for Ki67 proliferation marker and TUNEL assay for apoptosis detection in WT and RR HK-1 tumors. Data presented as mean ± SD, *n* ≥ 5. *, *P* < 0.05; **, *P* < 0.01; ***, *P* < 0.001. **e** mRNA expression of HDAC6 after Pano treatment in WT and RR FaDu, WT and RR HK-1 tumors. Fold-change is calculated against the respective WT control. Data presented as mean ± SD, *n* ≥ 5. *, *P* < 0.05; **, *P* < 0.01.)

Additional file 4 (Fig. S4 Target validation screen using Class I or II selective HDAC inhibitors. **a** Single drug and combination dose response curves of AZD7762 with either Romidepsin (Class I selective HDAC inhibitor) or Roybinostat (Class II selective inhibitor) across (i) WT and (ii) RR FaDu, with accompanying **b** F_a_-CI (fraction affected-combination index) plot. **c** Single drug and combination dose response curves of AZD7762 with either Romidepsin or Roybinostat across (i) WT and (ii) RR HK-1, with accompanying **d** F_a_-CI plot. Combination indices of log(CI) <0 across a range of effect sizes (F_a_) is indicative of a synergistic interaction. Data presented as means ± SD of two technical replicates.)

Additional file 5 (Fig. S5 Class II selective HDAC inhibitor preferentially targets RR-HNC models. Representative immunoblots of histone and non-histone proteins regulated by HDACs in WT and RR **a** FaDu and **b** HK-1 treated singly with Romidepsin (class I selective HDACi) or Roybinostat (class II selective HDACi), or in combination with AZD7762 for 48h.)

Additional file 6 (Fig. S6 RR-specific synergy is largely attributed to HDAC6 targeting. Dose-response curves of AZD7762 treated singly or in combination with siRNAs targeting HDAC1, HDAC2, HDAC4 or HDAC6 transcripts in WT (blue) or RR (pink) HK-1.)

Additional file 7 (Fig. S7 Knockdown of HDAC1/2/4 does not phenocopy panobinostat-mediated treatment response in RR cells. Representative immunoblots of proteins involved in the DNA damage regulatory system and cell cycle pathway in WT and RR FaDu treated singly with siRNAs targeting **a** HDAC1, **b** HDAC2, or **c** HDAC4 or in combination for 48h.)

Additional file 8 (Fig. S8 Analysis of SP1 DNA binding activity in WT and RR FaDu. Competition and knockdown assays to determine binding specificity of SP1 to its consensus sequence. C1 and C2 are resulting DNA-protein complexes formed.)

Additional file 9 (Fig. S9 SP1 ChIP-qPCR assay in WT and RR HK-1 cells. ChIP and qPCR analysis of SP1 binding levels at *MYC*, *RAD51* or *FOXM1* activating promoter regions in WT and RR HK-1 cells. Normal rabbit IgG antibody was used as a control for non-specific binding in ChIP assays.)

Additional file 10 (Fig. S10 Comparative transcriptomic profiling of isogenic RR models treated with or without siHDAC6. **a** Correlation heatmap of 24 samples from triplicated experiment in FaDu and HK-1 models. The differences between treatment type were larger than the cell phenotypes. **b** Volcano plot of differentially expressed (DE) genes identified from siNC-RR compared to siNC-WT cells. **c** Volcano plot of differentially expressed (DE) genes identified from siHDAC6-RR compared to siNC-RR cells. **d** Sensitivity analysis of the output from gene selection refinement process in both FaDu and HK-1, and common genes identified from intersection analysis of both cell lines.)

Additional file 11 (Fig. S11 Consensus gene panel and pathway analysis. **a** Panel of 24 genes differentially expressed in siNC-RR cells without treatment after refinement process. The magnitude of change in RR cells were higher than WT cells. Green, FaDu cell line; Pink, HK-1 cell line. **b** Geneset enrichment analysis (GSEA) with Gene ontology (GO) library depicting top 10 pathways significantly enriched in panel of 24 genes following refinement process.)

Additional file 12.

## Data Availability

Datasets generated and analyzed in this study are included in this published article and its supplementary information files. Raw data and materials are available from the corresponding authors on reasonable request.

## Abbreviations

HNC: Head and neck cancer
RT: Radiotherapy
RR: Radioresistant
QPOP: Quadratic Phenotypic Optimization Platform
HDAC: Histone deacetylase
EGFR: Epidermal growth factor receptor
DDR: DNA damage response
IC_50_: Half-maximal inhibitory concentrations
OACD: Orthogonal array composite design
CI: Combination index
GSEA: Gene set enrichment analysis
GO: Gene Ontology
