# Supplementary figures and images for: Combinatorial functionomics identifies HDAC6-dependent molecular vulnerability of radioresistant head and neck cancer
Source: Exp Hematol Oncol. 2025 Jan 12;14:5. doi: 10.1186/s40164-024-00590-8 (PMC11727331; doi:10.1186/s40164-024-00590-8)

A

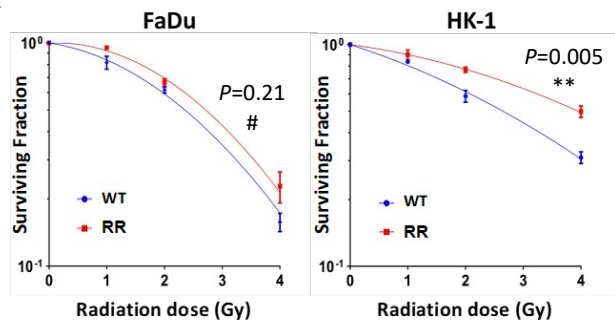

B

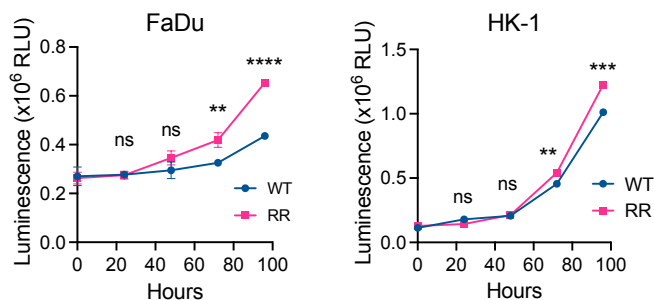

C i

WT FaDu RR FaDu

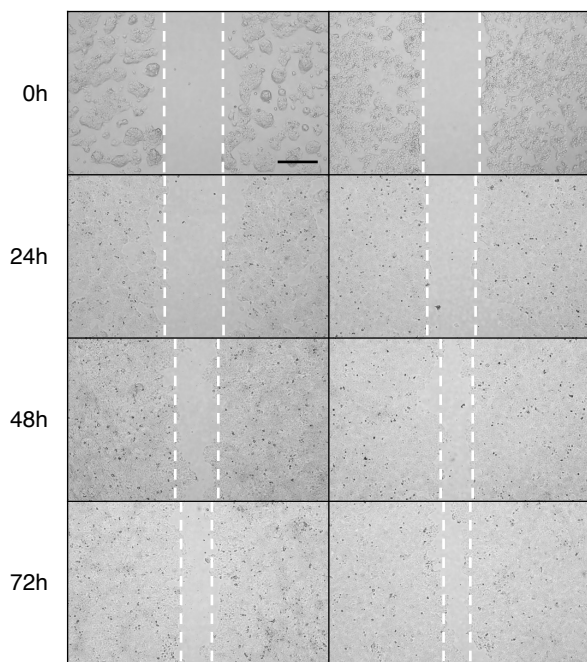

ii

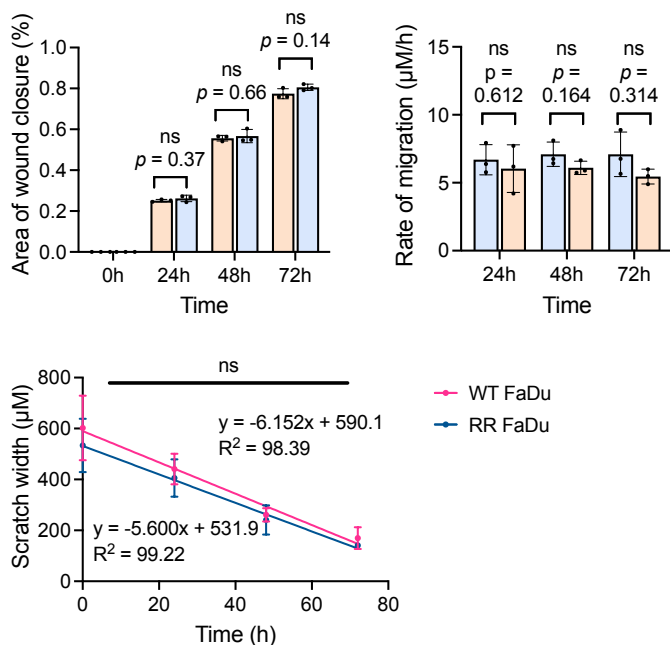

D i

WT HK-1 RR HK-1

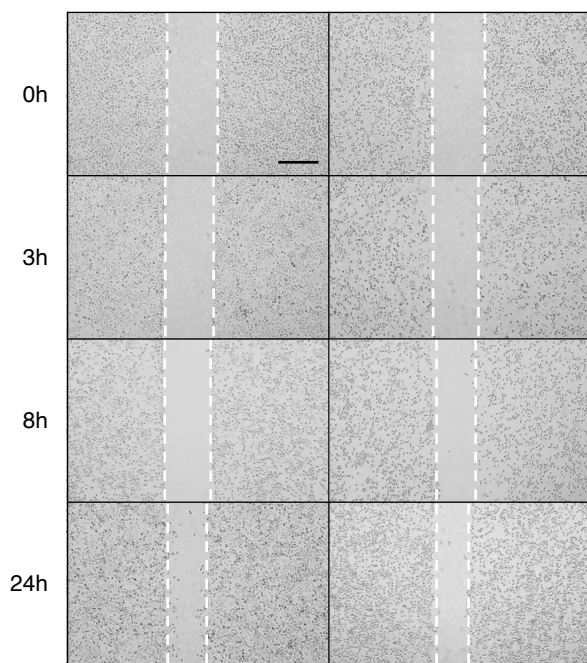

ii

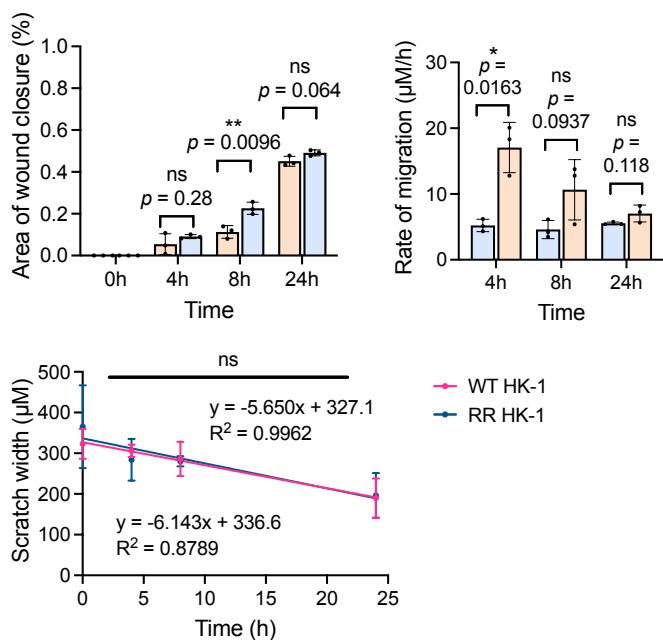

Supplement: Supplementary file 1 — Additional file 1 (Fig. S1 Characterization of in vitro isogenic models of radioresistance generated from serial irradiation method. a Survival curve of WT and RR FaDu and HK-1 cells following increasing doses of 0 to 4 Gy X-irradiation. b Growth curve of WT and RR FaDu and HK-1 cells derived from CTG luminescence values taken at the indicated timepoints. c Wound scratch assay performed on WT and RR FaDu cells with (i) representative images and accompanying quantification of (ii) area of wound closure (%), rate of migration (μM/h) and scratch width (μM). d Wound scratch assay performed on WT and RR HK-1 cells with (i) representative images and accompanying quantification of (ii) area of wound closure (%), rate of migration (μM/h) and scratch width (μM). All data presented as mean ± SD of three biological replicates. *, P < 0.05; **, P < 0.01; ***, P < 0.001; ****, P < 0.0001. All statistical analyses were performed using two-tailed Student’s t test.) [file 40164_2024_590_MOESM1_ESM.pdf]

A i

WT FaDu

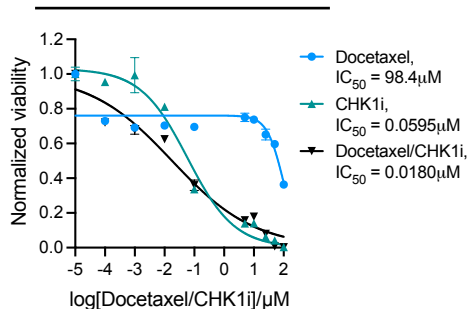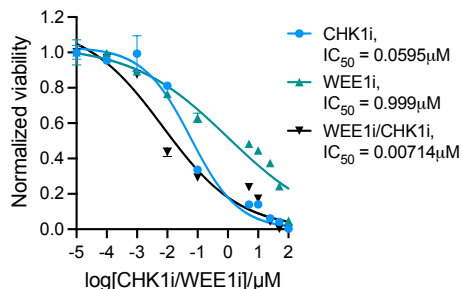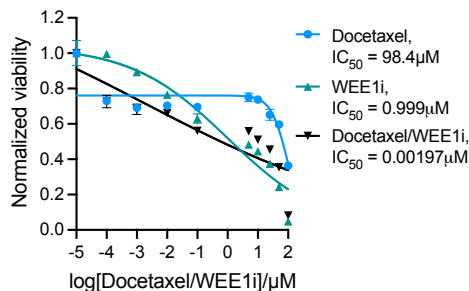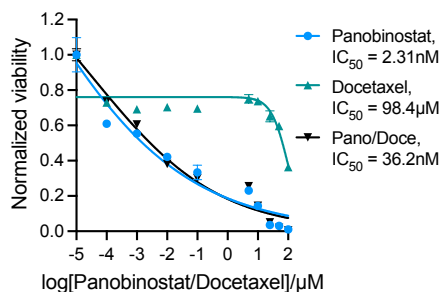

ii

RR FaDu

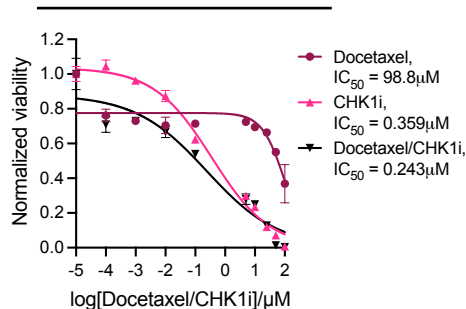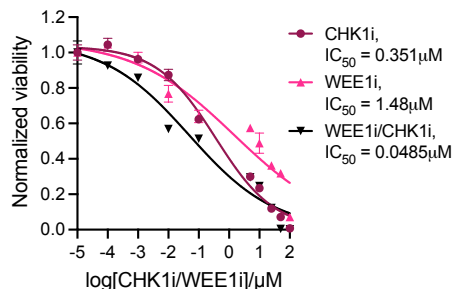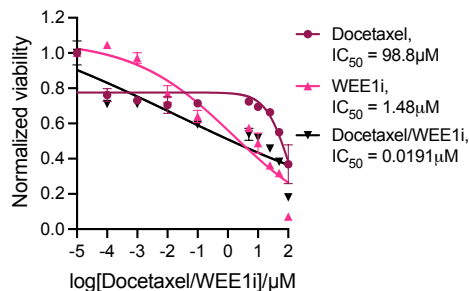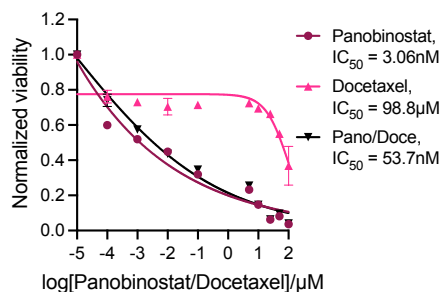

B

Docetaxel + CHK1i

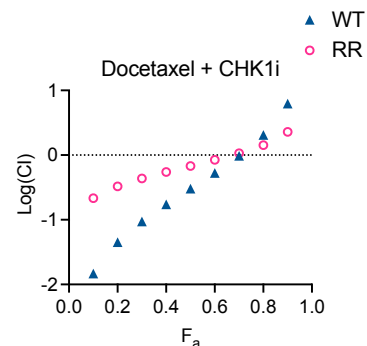

CHK1i + WEE1i

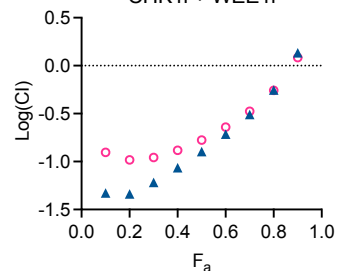

Docetaxel + WEE1i

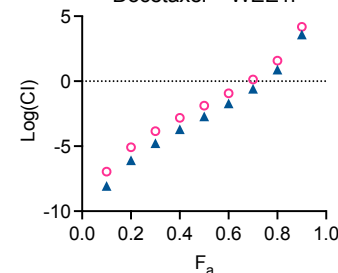

Panobinostat + Docetaxel

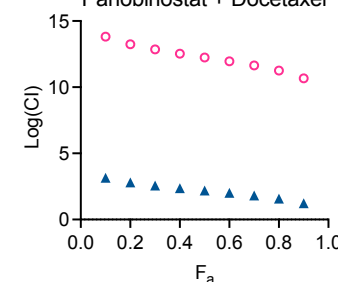

Supplement: Supplementary file 2 — Additional file 2 (Fig. S2 Validation screen of top five QPOP-ranked drug combinations. a Single drug and combination dose response curves of QPOP-derived top-ranking drug pairs across (i) WT and (ii) RR FaDu, with accompanying b Fa-CI (fraction affected-combination index) plot. Combination indices of log(CI) <0 across a range of effect sizes (Fa) is indicative of a synergistic interaction. Data presented as means ± SD of two technical replicates.) [file 40164_2024_590_MOESM2_ESM.pdf]

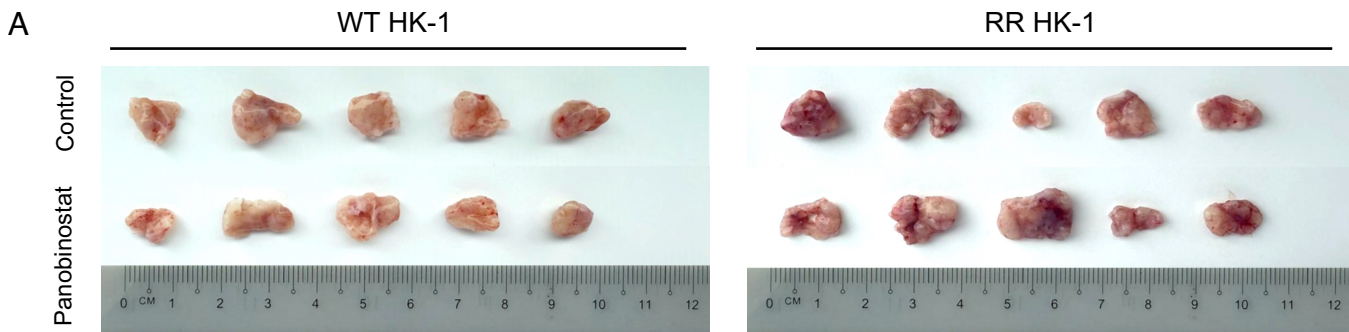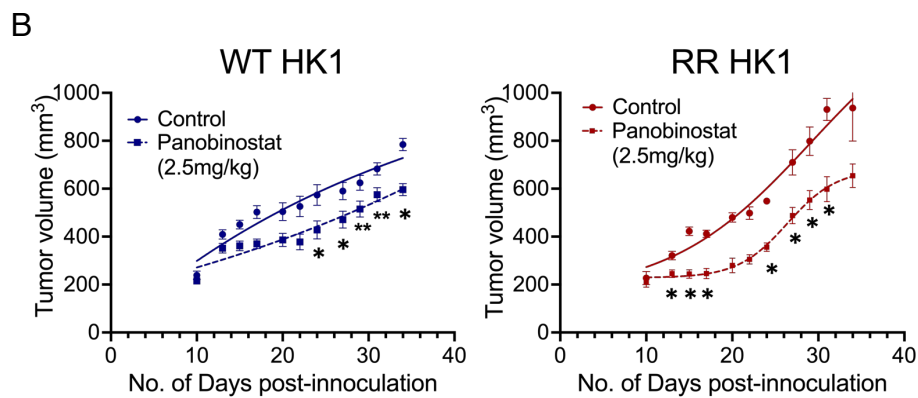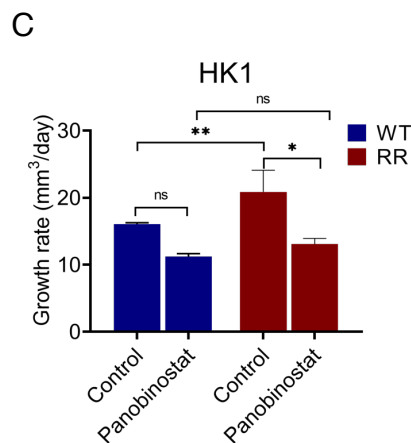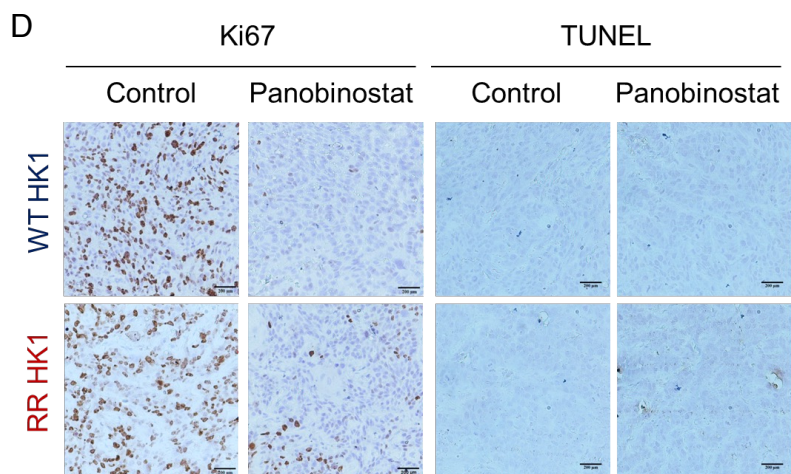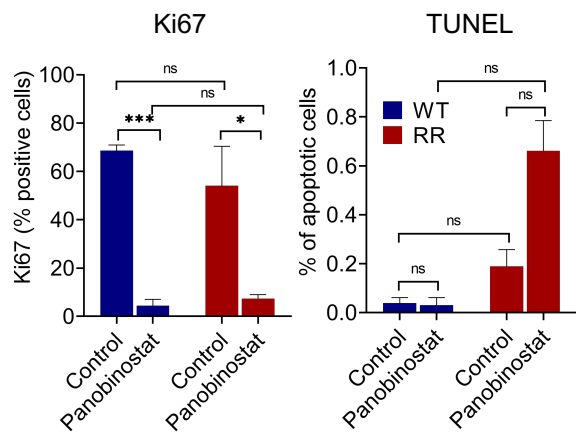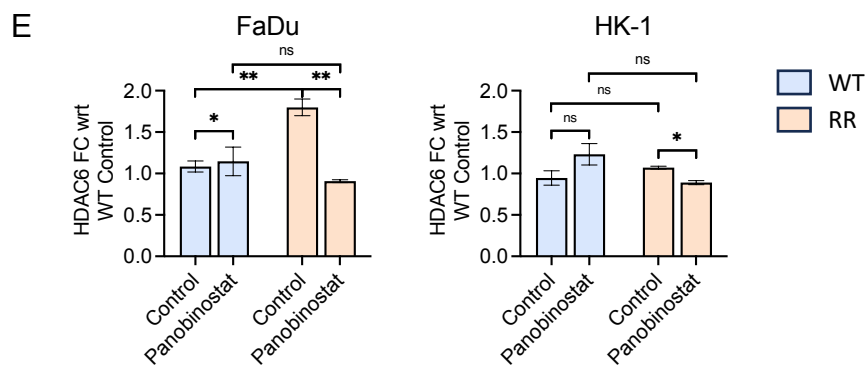

Supplement: Supplementary file 3 — Additional file 3 (Fig. S3 Panobinostat exhibits anti-tumors effects in in vivo models of RR-HNC. a Representative tumor images, and b tumor growth curve of WT and RR HK-1 during treatment for 34 days with vehicle control or 2.5 mg/kg Pano. Data presented as mean ± SD, n ≥ 5. *, P < 0.05. c Growth rate of WT and RR HK-1 tumors vehicle control and treated tumors at treatment end-point. Data presented as mean ± SD, n ≥ 5. *, P < 0.05; **, P < 0.01. d Immunohistochemistry (IHC) analysis for Ki67 proliferation marker and TUNEL assay for apoptosis detection in WT and RR HK-1 tumors. Data presented as mean ± SD, n ≥ 5. *, P < 0.05; **, P < 0.01; ***, P < 0.001. e mRNA expression of HDAC6 after Pano treatment in WT and RR FaDu, WT and RR HK-1 tumors. Fold-change is calculated against the respective WT control. Data presented as mean ± SD, n ≥ 5. *, P < 0.05; **, P < 0.01.) [file 40164_2024_590_MOESM3_ESM.pdf]

A

WT FaDu

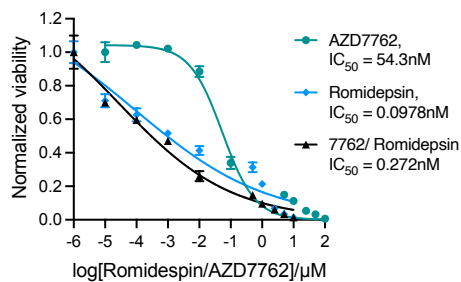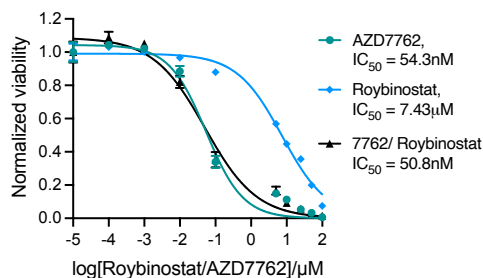

ii

RR FaDu

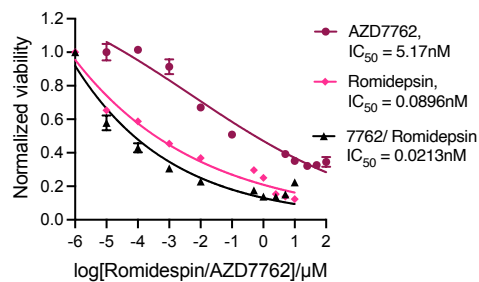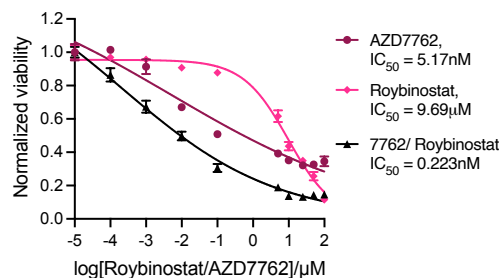

B

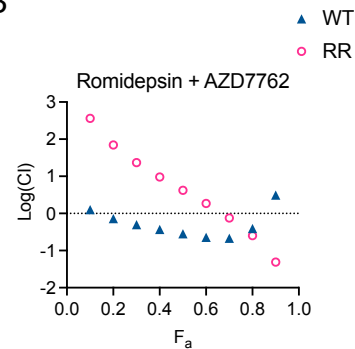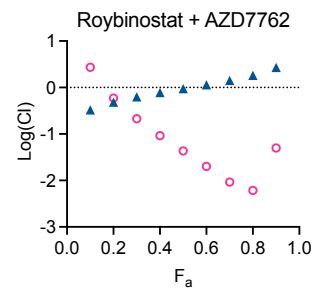

C

WT HK-1

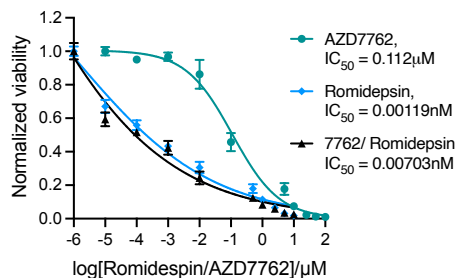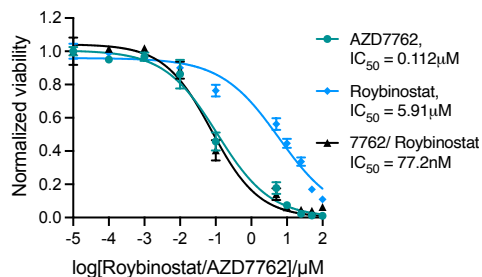

ii

RR HK-1

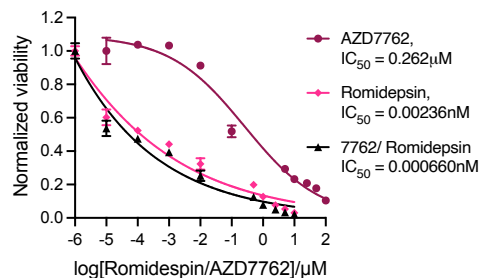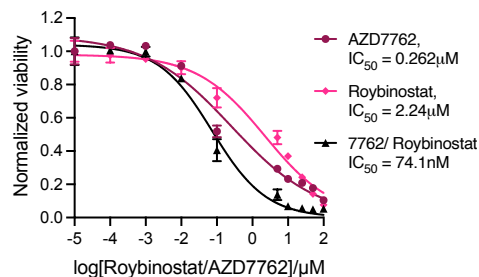

D

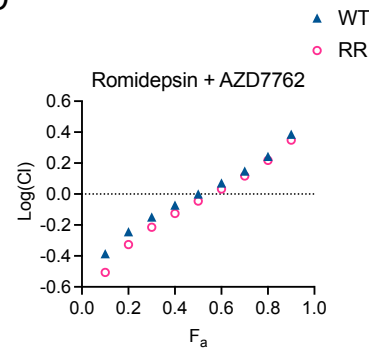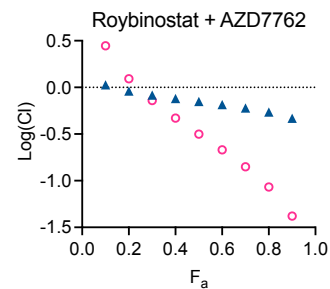

Supplement: Supplementary file 4 — Additional file 4 (Fig. S4 Target validation screen using Class I or II selective HDAC inhibitors. a Single drug and combination dose response curves of AZD7762 with either Romidepsin (Class I selective HDAC inhibitor) or Roybinostat (Class II selective inhibitor) across (i) WT and (ii) RR FaDu, with accompanying b Fa-CI (fraction affected-combination index) plot. c Single drug and combination dose response curves of AZD7762 with either Romidepsin or Roybinostat across (i) WT and (ii) RR HK-1, with accompanying d Fa-CI plot. Combination indices of log(CI) <0 across a range of effect sizes (Fa) is indicative of a synergistic interaction. Data presented as means ± SD of two technical replicates.) [file 40164_2024_590_MOESM4_ESM.pdf]

**A**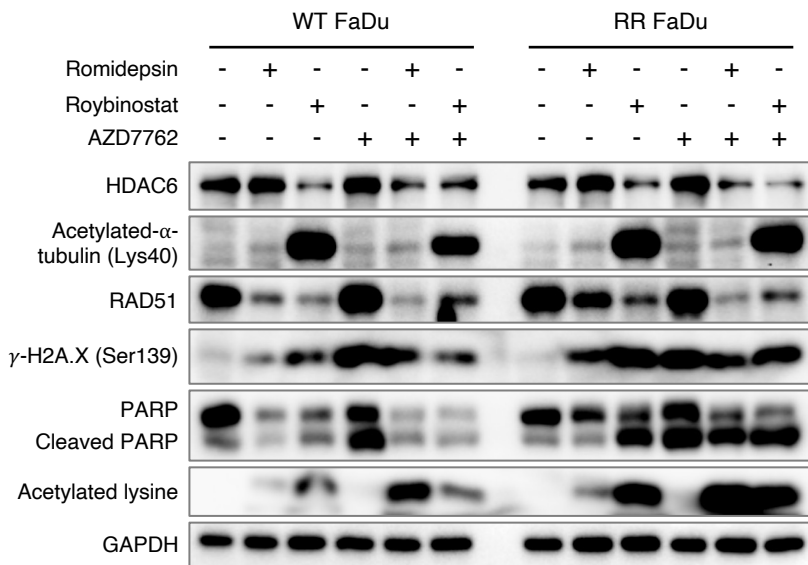**B**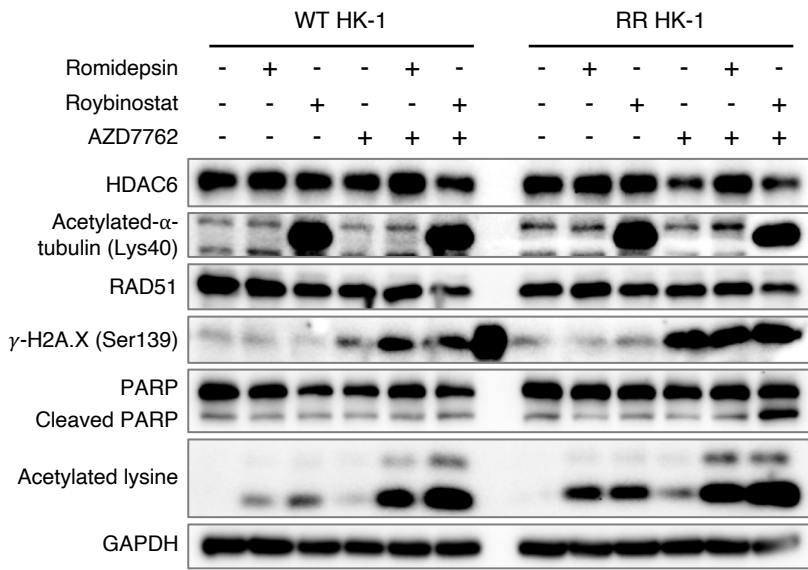

Supplement: Supplementary file 5 — Additional file 5 (Fig. S5 Class II selective HDAC inhibitor preferentially targets RR-HNC models. Representative immunoblots of histone and non-histone proteins regulated by HDACs in WT and RR a FaDu and b HK-1 treated singly with Romidepsin (class I selective HDACi) or Roybinostat (class II selective HDACi), or in combination with AZD7762 for 48h.) [file 40164_2024_590_MOESM5_ESM.pdf]

**A**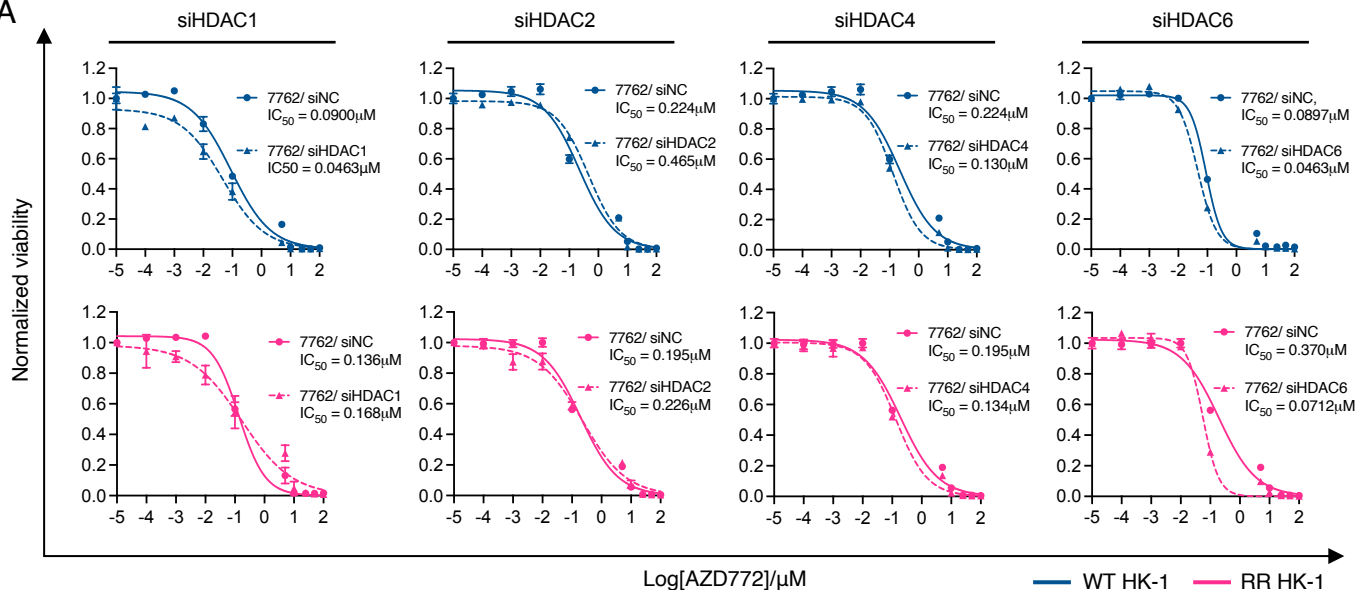**B**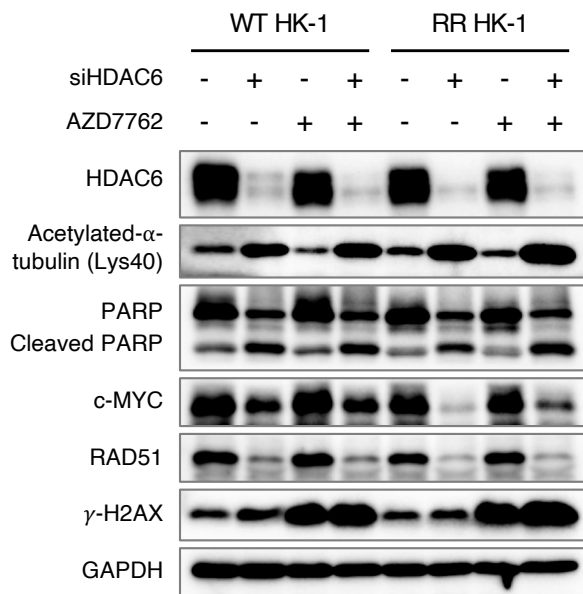

Supplement: Supplementary file 6 — Additional file 6 (Fig. S6 RR-specific synergy is largely attributed to HDAC6 targeting. Dose-response curves of AZD7762 treated singly or in combination with siRNAs targeting HDAC1, HDAC2, HDAC4 or HDAC6 transcripts in WT (blue) or RR (pink) HK-1.) [file 40164_2024_590_MOESM6_ESM.pdf]

**A**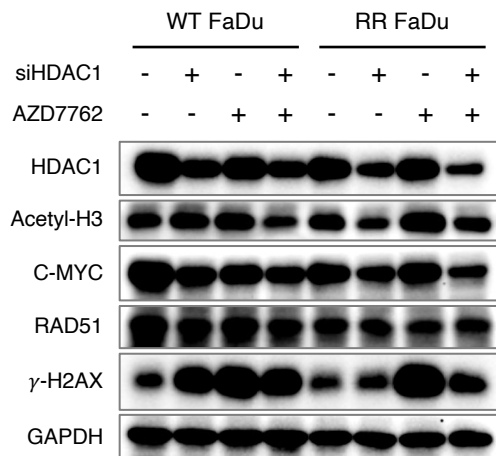**B**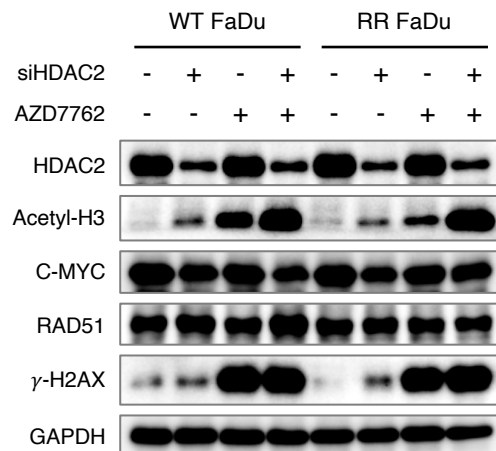**C**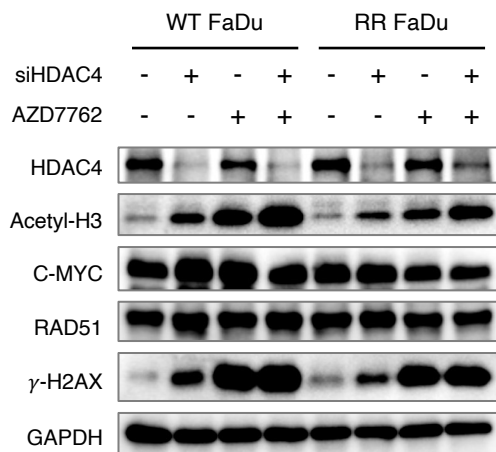

Supplement: Supplementary file 7 — Additional file 7 (Fig. S7 Knockdown of HDAC1/2/4 does not phenocopy panobinostat-mediated treatment response in RR cells. Representative immunoblots of proteins involved in the DNA damage regulatory system and cell cycle pathway in WT and RR FaDu treated singly with siRNAs targeting a HDAC1, b HDAC2, or c HDAC4 or in combination for 48h.) [file 40164_2024_590_MOESM7_ESM.pdf]

|            |   | WT FaDu |   |   | RR FaDu |   |   |
|------------|---|---------|---|---|---------|---|---|
| Hot probe  | + | +       | + | + | +       | + | + |
| Cold probe | - | -       | + | - | -       | + | - |
| si-NC      | - | +       | + | - | +       | + | - |
| si-SP1     | - | -       | - | + | -       | - | + |

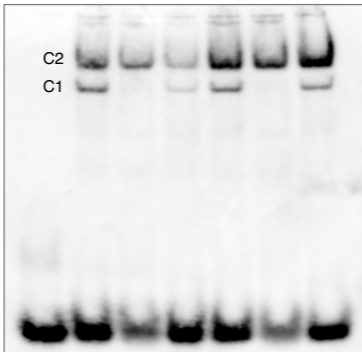

Supplement: Supplementary file 8 — Additional file 8 (Fig. S8 Analysis of SP1 DNA binding activity in WT and RR FaDu. Competition and knockdown assays to determine binding specificity of SP1 to its consensus sequence. C1 and C2 are resulting DNA-protein complexes formed.) [file 40164_2024_590_MOESM8_ESM.pdf]

*MYC*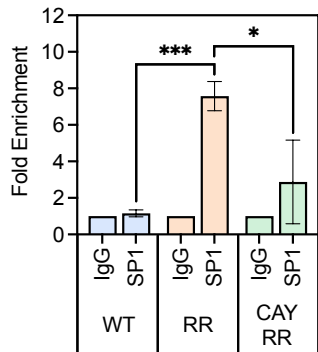*RAD51*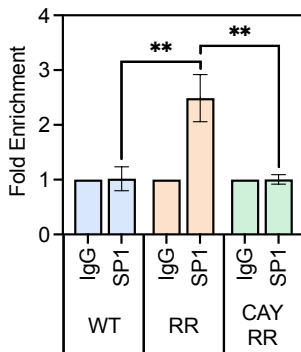*FOXO1*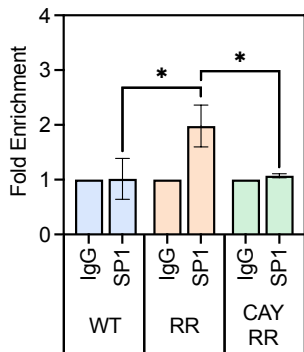

WT HK-1

RR HK-1

100nM CAY10603 RR HK-1

Supplement: Supplementary file 9 — Additional file 9 (Fig. S9 SP1 ChIP-qPCR assay in WT and RR HK-1 cells. ChIP and qPCR analysis of SP1 binding levels at MYC, RAD51 or FOXM1 activating promoter regions in WT and RR HK-1 cells. Normal rabbit IgG antibody was used as a control for non-specific binding in ChIP assays.) [file 40164_2024_590_MOESM9_ESM.pdf]

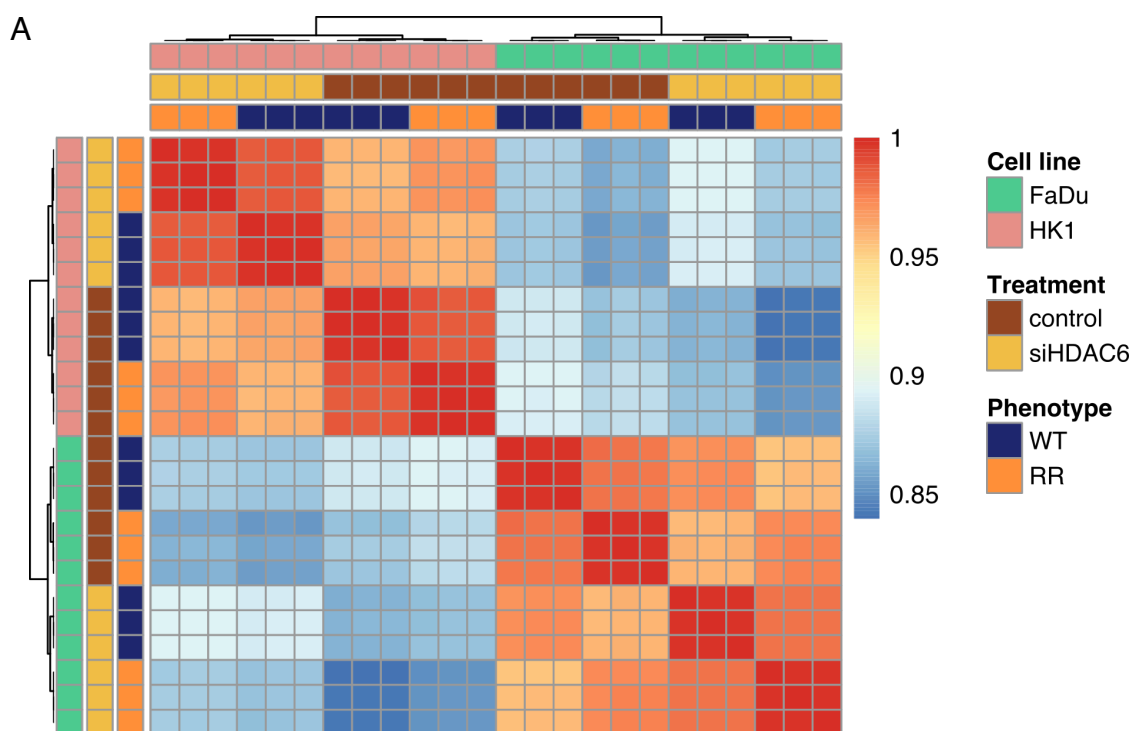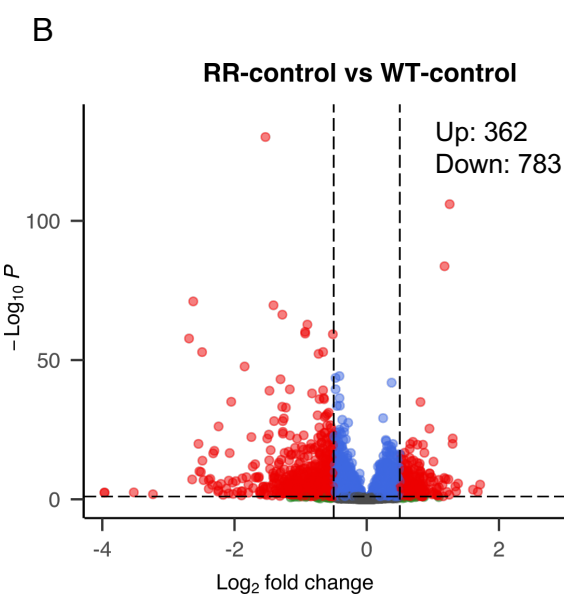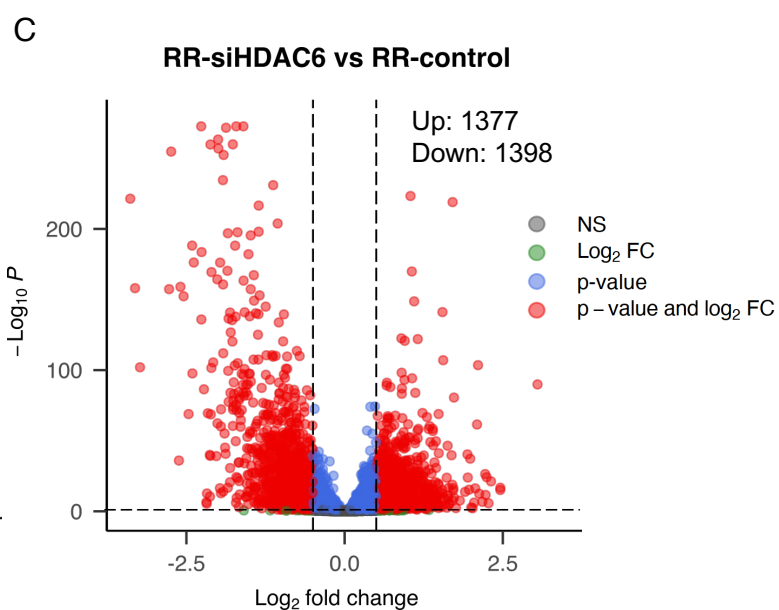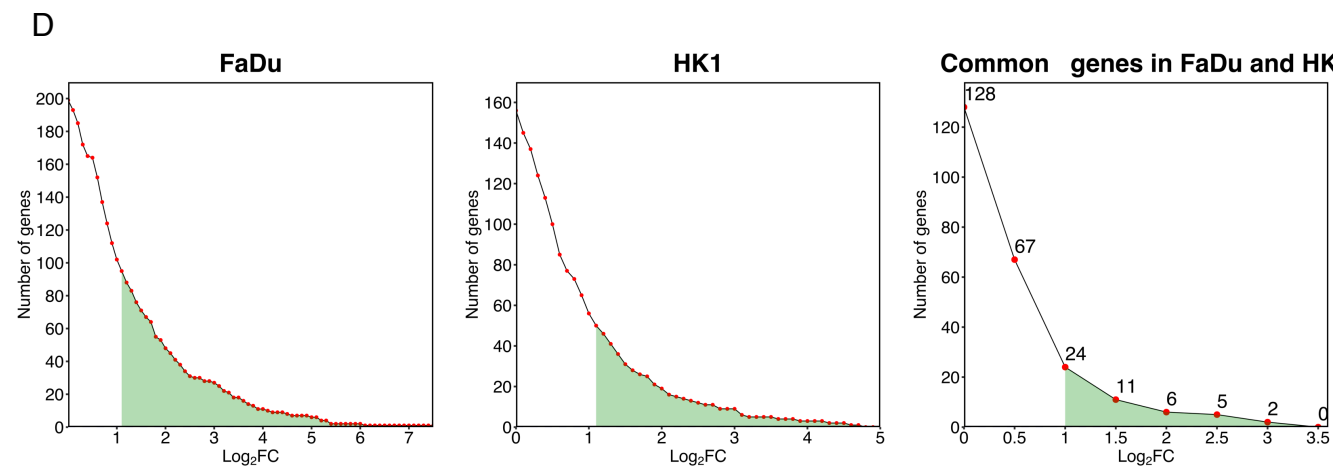

Supplement: Supplementary file 10 — Additional file 10 (Fig. S10 Comparative transcriptomic profiling of isogenic RR models treated with or without siHDAC6. a Correlation heatmap of 24 samples from triplicated experiment in FaDu and HK-1 models. The differences between treatment type were larger than the cell phenotypes. b Volcano plot of differentially expressed (DE) genes identified from siNC-RR compared to siNC-WT cells. c Volcano plot of differentially expressed (DE) genes identified from siHDAC6-RR compared to siNC-RR cells. d Sensitivity analysis of the output from gene selection refinement process in both FaDu and HK-1, and common genes identified from intersection analysis of both cell lines.) [file 40164_2024_590_MOESM10_ESM.pdf]

A

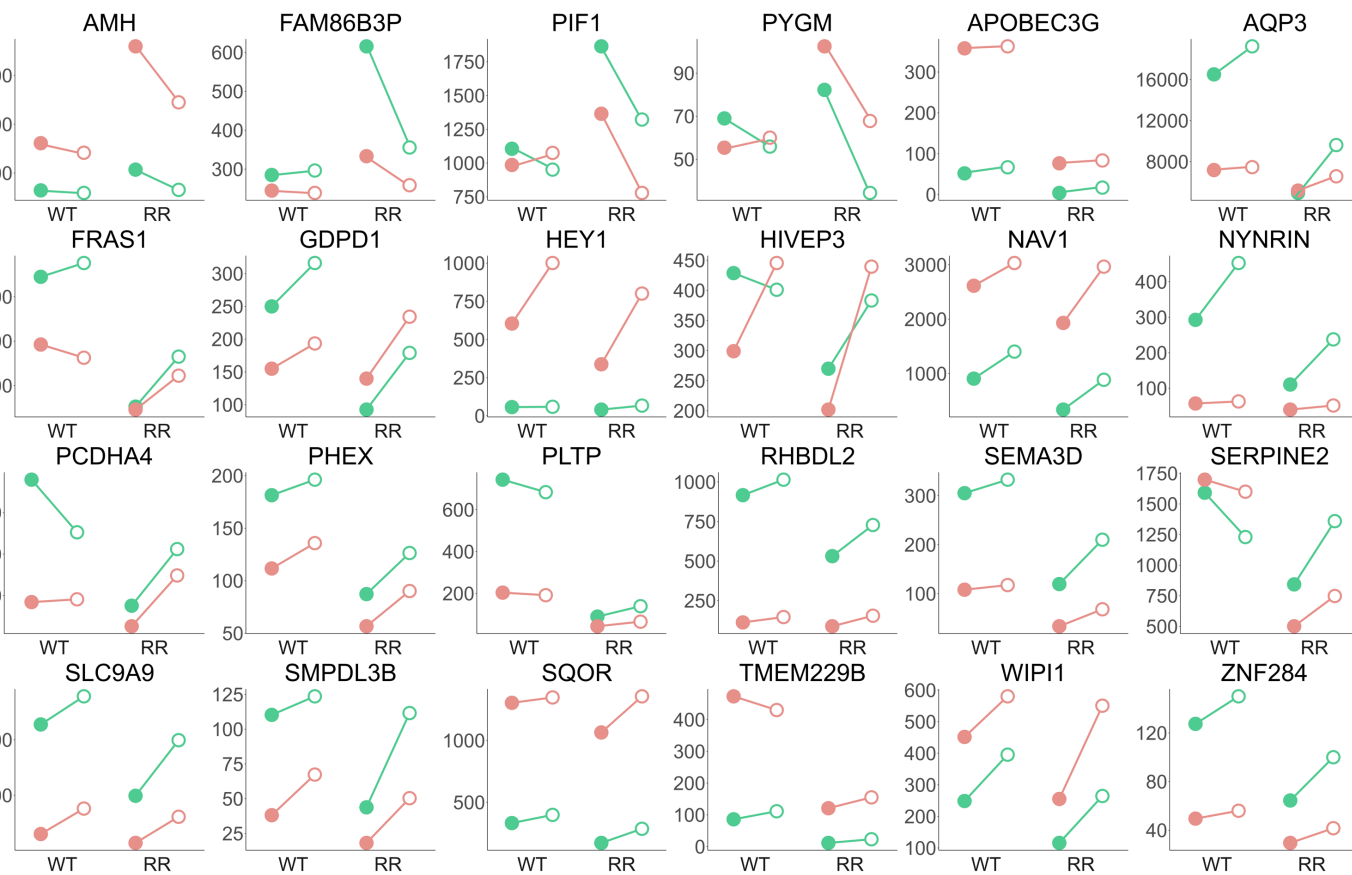

B

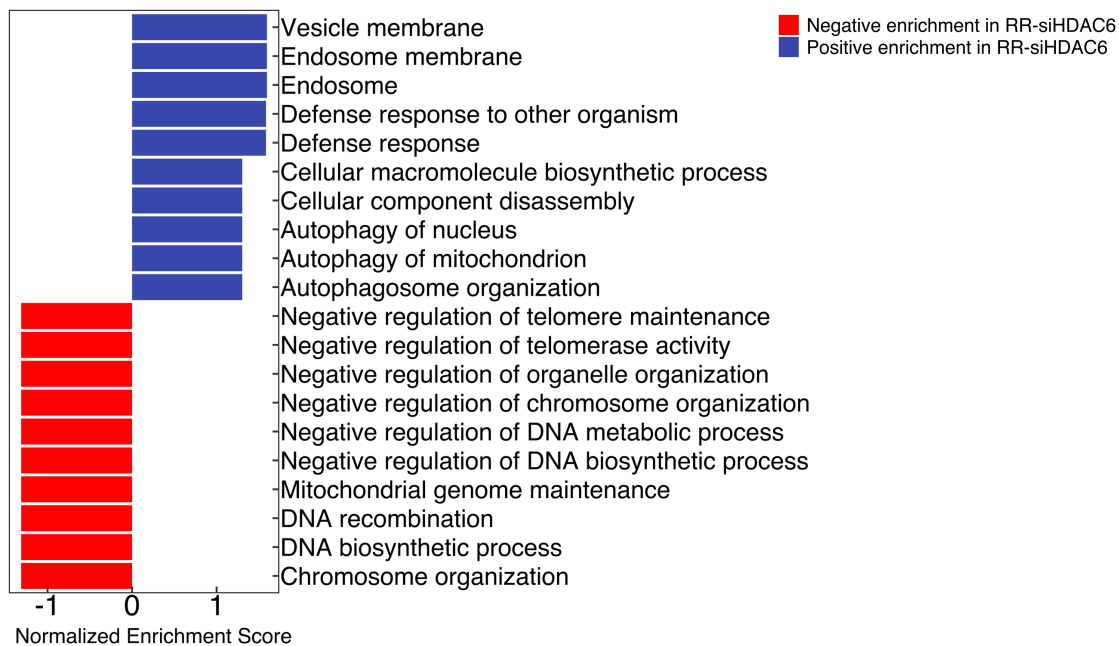

Supplement: Supplementary file 11 — Additional file 11 (Fig. S11 Consensus gene panel and pathway analysis. a Panel of 24 genes differentially expressed in siNC-RR cells without treatment after refinement process. The magnitude of change in RR cells were higher than WT cells. Green, FaDu cell line; Pink, HK-1 cell line. b Geneset enrichment analysis (GSEA) with Gene ontology (GO) library depicting top 10 pathways significantly enriched in panel of 24 genes following refinement process.) [file 40164_2024_590_MOESM11_ESM.pdf]
