# Supplementary material for: Combinatorial functionomics identifies HDAC6-dependent molecular vulnerability of radioresistant head and neck cancer
Source: Exp Hematol Oncol. 2025 Jan 12;14:5. doi: 10.1186/s40164-024-00590-8 (PMC11727331; doi:10.1186/s40164-024-00590-8)
Supplement: Supplementary file 12 — Additional file 12. [file 40164_2024_590_MOESM12_ESM.docx]

**SUPPLEMENTARY INFORMATION**

**Table S1: Primer sequences for SP1 ChIP-qPCR analysis.**

| Gene | Direction (5’ 🡪 3’) | Primer sequence |
| --- | --- | --- |
| *MYC* | Forward | AGGGCTTCTCAGAGGCTTG |
|  | Reverse | CCTATTCGCTCCGGATCTC |
| *RAD51* | Forward | CTGTAAACTCGCGCAGGATC |
|  | Reverse | GCTTTCAGAATTCCCGCCAA |
| *FOXM1* | Forward | CACCTCAGCCTCCAGAGTAG |
|  | Reverse | TAAACAAATGTGGGCTGGGC |
